# Assessing the potential of deep learning for protein–ligand docking

**DOI:** 10.1038/s42256-025-01160-1

**Published:** 2025-12-31

**Authors:** Alex Morehead, Nabin Giri, Jian Liu, Pawan Neupane, Jianlin Cheng

**Affiliations:** 1 Lawrence Berkeley National Laboratory, Berkeley, CA USA; 2 Electrical Engineering and Computer Science, NextGen Precision Health, University of Missouri, Columbia, MO USA

**Keywords:** Computational science, Machine learning, Software, Computer science, Protein structure predictions

## Abstract

The effects of ligand binding on protein structures and their in vivo functions carry numerous implications for modern biomedical research and biotechnology development efforts such as drug discovery. Although several deep learning (DL) methods and benchmarks designed for protein–ligand docking have recently been introduced, so far no previous works have systematically studied the behaviour of the latest docking and structure prediction methods within the broadly applicable context of: (1) using predicted (apo) protein structures for docking (for example, for applicability to new proteins); (2) binding multiple (cofactor) ligands concurrently to a given target protein (for example, for enzyme design); and (3) having no previous knowledge of binding pockets (for example, for generalization to unknown pockets). To enable a deeper understanding of the real-world utility of docking methods, we introduce PoseBench, a comprehensive benchmark for broadly applicable protein–ligand docking. PoseBench enables researchers to rigorously and systematically evaluate DL methods for apo-to-holo protein–ligand docking and protein–ligand structure prediction using both primary ligand and multiligand benchmark datasets, the latter of which we introduce to the DL community. Empirically, using PoseBench, we find that: (1) DL cofolding methods generally outperform comparable conventional and DL docking baseline algorithms, but popular methods such as AlphaFold 3 are still challenged by prediction targets with new protein–ligand binding poses; (2) certain DL cofolding methods are highly sensitive to their input multiple sequence alignments, whereas others are not; and (3) DL methods struggle to strike a balance between structural accuracy and chemical specificity when predicting new or multiligand protein targets.

## Abstract (web summary)

Morehead et al. introduce the benchmark PoseBench and evaluate the strengths and limitations of current AI-based protein–ligand docking and structure prediction methods.

## Main

The field of drug discovery has long been challenged with a critical task: determining the structure of ligand molecules in complex with proteins and other key biomolecules^1^. As accurately identifying such complex structures (in particular multiligand structures) can yield advanced insights into the binding dynamics and functional characteristics (and thereby, the medicinal potential) of numerous protein complexes in vivo, in recent years, substantial resources have been spent developing new experimental and computational techniques for protein–ligand structure determination^2^. Over the last decade, machine learning (ML) methods for structure prediction have become indispensable components of modern structure determination at scale, with AlphaFold 2 for protein structure prediction being a hallmark example^3,4^.

As the field has gradually begun to investigate whether proteins in complex with other types of molecules can faithfully be modelled with ML (and particularly deep learning (DL)) techniques^5–7^, several new works in this direction have suggested the promising potential of such approaches to protein–ligand structure determination^8–11^. Nonetheless, it remains to be shown the extent to which the latest of such (docking and cofolding-based) DL methods can adequately generalize to the context of binding new or uncommon protein–ligand interaction (PLI) pockets and multiple interacting ligand molecules (which, for example, can alter the chemical functions of various enzymes) as well as whether such methods can faithfully model amino acid-specific types of PLIs natively found in crystallized biomolecular structures.

To bridge this knowledge gap, we introduce a unified benchmark for protein–ligand docking and structure prediction that evaluates the performance of several recent DL-based baseline methods (DiffDock-L, DynamicBind, NeuralPLexer, RoseTTAFold-All-Atom, Chai-1, Boltz-1 and AlphaFold 3) as well as conventional algorithms (P2Rank + AutoDock Vina) for primary and multiligand docking, which suggests that DL cofolding methods generally outperform conventional algorithms but remain challenged by new or uncommon prediction targets.

In contrast with several recent works using crystal protein structures for protein–ligand docking^12,13^, the docking benchmark results that we present in this work are all within the context of standardized input multiple sequence alignments (MSAs) and high accuracy apo-like (that is, AlphaFold 3-predicted) protein structures (Supplementary Appendix D) without specifying known binding pockets, which notably enhances the broad applicability of this study’s findings.

Our newly proposed benchmark, PoseBench, enables specific insights into necessary areas of future work for accurate and generalizable biomolecular structure prediction, including that DL methods struggle to balance faithful modelling of native PLI fingerprints (PLIFs) with structural accuracy during pose prediction and that some DL cofolding methods (AlphaFold 3) are more dependent than others (Boltz-1 and Chai-1) on the availability of input MSAs.

Our benchmark results also highlight the importance of including challenging (out-of-distribution) datasets when evaluating future DL methods and measuring their ability to recapitulate amino acid-specific PLIFs with an appropriate new metric that we introduce in this work.

## Related work

### Structure prediction of PLI complexes

The field of DL-driven protein–ligand structure determination was largely sparked with the development of geometric DL methods such as EquiBind^14^ and TANKBind^15^ for direct (that is, regression-based) prediction of bound ligand structures in protein complexes. Notably, these predictive methods could estimate localized ligand structures in complex with multiple protein chains as well as the associated complexes’ binding affinities. However, in addition to their limited predictive accuracy, they have more recently been found to frequently produce steric clashes between protein and ligand atoms, notably hindering their widespread adoption in modern drug discovery pipelines.

### Protein–ligand structure prediction and docking

Shortly following the first wave of predictive methods for protein–ligand structure determination, DL methods such as DiffDock^8^ demonstrated the utility of a new approach to this problem by reframing protein–ligand docking as a generative modelling task, whereby multiple ligand conformations can be generated for a particular protein target and rank-ordered using a predicted confidence score^16^. This approach has inspired many follow-up works offering alternative formulations of this generative approach to the problem^7,9–11,13,17–33^, with some of such follow-up works also being capable of accurately modelling protein flexibility upon ligand binding or predicting binding affinities to a high degree of accuracy.

### Benchmarking efforts for protein–ligand complexes

In response to the large number of new methods that have been developed for protein–ligand structure prediction, recent works have introduced several new datasets and metrics with which to evaluate newly developed methods^34^, with some of such benchmarking efforts focusing on modelling single-ligand protein interactions^12,35–40^ and others specializing in the assessment of multiligand protein interactions^41^. One of the motivations for introducing PoseBench in this work is to bridge this gap by systematically assessing a selection of the latest (pocket-blind) structure prediction methods within both interaction regimes, using unbound (apo) protein structures with docking methods and challenging DL cofolding methods to predict full bioassemblies from primary sequences. Our benchmarking results in the following section demonstrate the relevance and utility of this comprehensive new evaluation suite for the future of protein–ligand modelling.

## Results and discussion

**Fig. 1:**
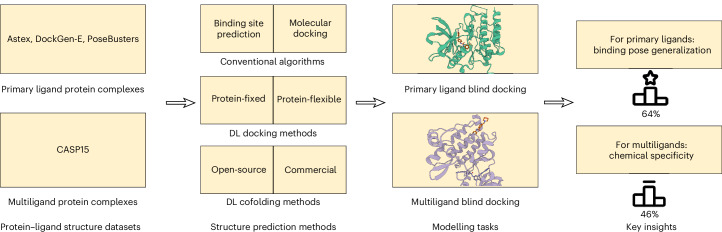
The PoseBench benchmark. Overview of PoseBench, our comprehensive benchmark for broadly applicable DL modelling of primary and multiligand protein complex structures. Baseline algorithms within the benchmark include a range of the latest DL docking and cofolding methods, both open-source and commercially restrictive, as well as conventional algorithms for docking. Key observations derived using PoseBench include the discontinuity between structure and interaction modelling performance for new or uncommon prediction targets and the heavy reliance of key DL cofolding methods on MSA-based input features to achieve high structural accuracy.

In this section, we present PoseBench’s results for primary and multiligand protein–ligand docking and structure prediction and discuss their implications for future work, as succinctly illustrated in Fig. 1. Note that, across all experiments, for generative methods, we report their performance metrics in terms of the mean and standard deviation across three independent runs of each method to gain insights into their interrun stability and consistency. Key metrics include a method’s percentage of structurally accurate ligand pose predictions with a (heavy atom centroid) root mean square deviation (r.m.s.d.) less than 2 (1) Å (that is, (c.)r.m.s.d. ≤ 2 (1) Å); its percentage of structurally accurate pose predictions that are also chemically valid according to the PoseBusters software suite (that is, r.m.s.d. ≤ 2 Å and PB-Valid), which can be affected by the posthoc application of structural relaxation driven by computationally expensive molecular dynamics (MD) simulations^42^ (that is, with relaxation); and our newly proposed Wasserstein matching (WM) score of its amino acid-specific predicted PLIFs (PLIF-WM). We formally define these metrics in ‘Metrics’. For interested readers, in Supplementary Appendix C, we report the average runtime and memory usage of each baseline method to determine which methods are the most efficient for real-world structure-based applications, and in Supplementary Appendix G we present Supplementary Results.

### Astex Diverse results

**Fig. 2:**
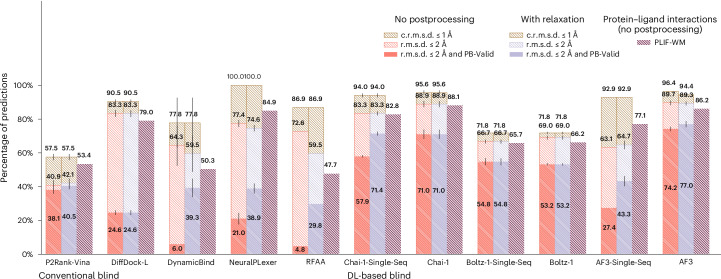
Astex Diverse results. Astex Diverse primary ligand docking success rates (*n* = 85 protein–ligand complexes). Data are presented as mean values ± s.d. over three independent predictions for each complex.

Containing PLI structures deposited in the RCSB Protein Data Bank (PDB)^43^ up until 2007, most of the well-known Astex Diverse dataset’s structures^44^ are present in the training data of each baseline method, but benchmarking results for this dataset (*n* = 85 protein–ligand complexes), shown in Fig. 2, indicate that only DL cofolding methods achieve higher structural and chemical accuracy rates (r.m.s.d. ≤ 2 Å and PB-Valid) than the conventional docking baseline AutoDock Vina combined with P2Rank for PLI binding site prediction to facilitate blind molecular docking. Interestingly, nearly all baseline methods identify the correct PLI binding pocket approximately 90% of the time, but only the DL cofolding methods Chai-1^30^, Boltz-1^31^ and AlphaFold 3 (AF3)^11^ achieve a reasonable balance between their rates of structural and chemical accuracy and chemical specificity (PLIF-WM), with the single-sequence (that is, MSA-ablated) version of AF3 being a notable exception. These results suggest that DL cofolding methods have learned the most comprehensive representations of this dataset’s input sequences, but only the DL cofolding method Chai-1 maintains strong performance without the availability of diverse input MSAs. One likely explanation for this phenomenon is that Chai-1’s training relied upon the availability of amino acid sequence embeddings generated by the protein language model ESM2^45^ in addition to features derived from input MSAs, which may have imbued the model with rich MSA-independent representations for biomolecular structure prediction.

### DockGen-E results

**Fig. 3:**
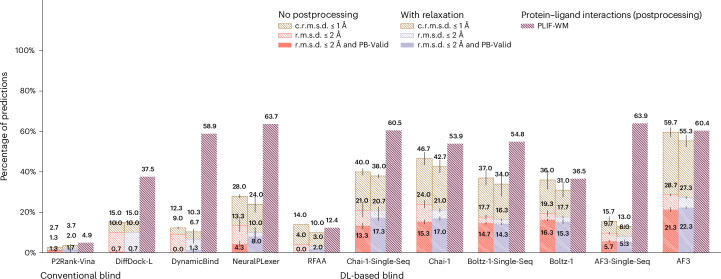
DockGen-E results. DockGen-E primary ligand docking success rates (*n* = 122 protein–ligand complexes). Data are presented as mean values ± s.d. over three independent predictions for each complex.

As visualized in Fig. 3, results with our new DockGen-E dataset of biologically relevant PLI complexes deposited in the PDB up to 2019 (*n* = 122 protein–ligand complexes) demonstrate that only the latest DL cofolding methods can locate a sizable fraction of structurally accurate PLI binding poses represented in this dataset. As such methods may have previously seen these PLI structures in their respective training data, it is surprising that even the latest AF3 model fails to identify a structurally and chemically accurate pose for more than 75% of the dataset’s complexes. Further, for Chai-1, Boltz-1 and AF3, their single-sequence variants achieve higher chemical specificity than their MSA-based versions, which may indicate that, for these methods, MSA features obfuscate primary sequence knowledge in favour of evolution-averaged (that is, amino acid-generic) representations. The overall lower range of PLIF-WM values achieved by each method for this dataset further suggests the increased chemical modelling difficulty of this dataset’s complexes compared with those presented by the Astex Diverse dataset. A potential source of these difficulties is that each of this dataset’s complexes represents a functionally distinct PLI binding pocket (as codified by ECOD domains^46^; see ref. ^47^ for more details) compared with data deposited in the PDB before 2019. As such, it is likely that Chai-1, Boltz-1 and AF3 are ‘overfitted’ to the most common types of PLI structures in the PDB and may overlook several uncommon types of PLI binding pockets present in nature.

### PoseBusters Benchmark results

**Fig. 4:**
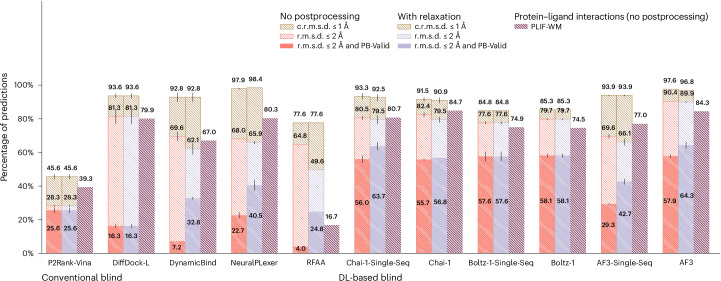
PoseBusters Benchmark results. PoseBusters Benchmark primary ligand docking success rates (*n* = 130/308 protein–ligand complexes). Data are presented as mean values ± s.d. over three independent predictions for each complex.

With approximately half of its PLI structures deposited in the PDB after AF3 and Boltz-1’s maximum-possible training data cutoff of 30 September, 2021 (*n* = 308 total protein–ligand complexes, filtered to *n* = 130 for subsequent analyses), the PoseBusters Benchmark dataset’s results, presented in Fig. 4, indicate once again that DL cofolding methods achieve top performance compared with conventional and DL-docking baseline methods. Nonetheless, we observed an interesting phenomenon whereby Chai-1 strikes a balance of structural and chemical accuracy and chemical specificity comparable with that of the best-performing AF3, even without input MSAs, potentially suggesting that Chai-1 achieves stronger binding pose generalization for this dataset than AF3. Moreover, with the single-sequence version of AF3, we again observed substantial degradations in its overall performance, whereas running Chai-1 with input MSAs achieved higher chemical specificity at the cost of marginal structural accuracy compared with running it in single-sequence mode. These observations highlight the importance in future work of carefully studying why and how the training of biomolecular structure generative models can be influenced to varying degrees by the availability and composition of diverse input MSAs.

### CASP15 results

**Fig. 5:**
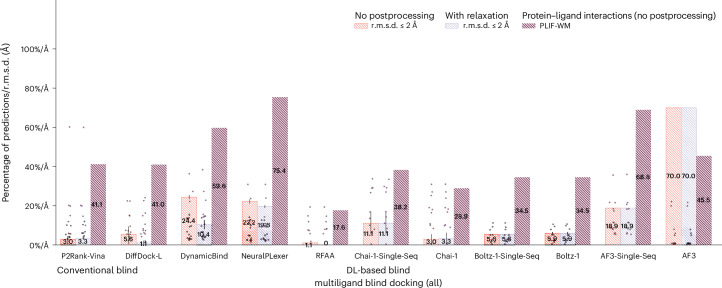
CASP15 multiligand results. CASP15 multiligand docking success rates (*n* = 13 protein–ligand complexes). Data are presented as mean values ± s.d. over three independent predictions for each complex.

**Extended Data Fig. 1:**
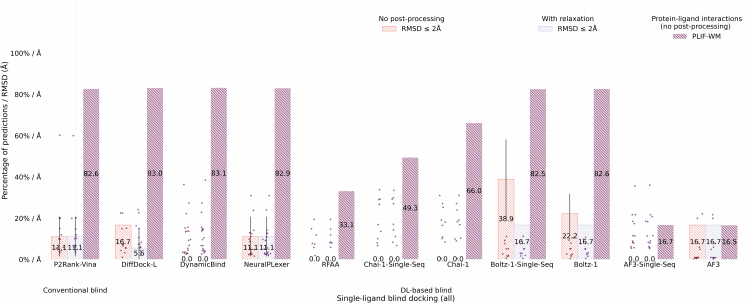
CASP15 Single-Ligand Results. CASP15 single-ligand docking success rates (n=6 protein-ligand complexes). Data are presented as mean values +/- standard deviations over three independent predictions for each complex.

As a new dataset of new and challenging PLI complexes on which no method has been trained, the CASP15 dataset’s multiligand results (*n* = 13 protein–ligand complexes), illustrated in Fig. 5, indicate that most methods fail to adequately generalize to multiligand prediction targets. However, AF3 stands out in this regard (only) when provided with input MSAs. As many of these CASP15 multiligand targets represent large, highly symmetrical protein complexes, it is likely that additional evolutionary information in the form of MSAs has improved AF3’s ability to predict higher-order protein–protein interactions for these targets. However, interestingly, its improved rate of structural accuracy comes at the cost of its protein–ligand chemical specificity (in comparison with its single-sequence results). For the CASP15 dataset’s single-ligand (that is, primary ligand) results (*n* = 6 protein–ligand complexes) presented in Extended Data Fig. 1, this trend is subverted in that conventional docking and open-source DL cofolding methods such as AutoDock Vina, NeuralPLexer and Boltz-1 outperform all other recent DL cofolding methods in modelling crystallized PLIFs while achieving comparable rates of structural accuracy. Given the small size of the CASP15 dataset, it is reasonable to conclude that DL methods, in particular some of the latest cofolding methods, may be challenged to predict protein–ligand complexes containing new PLIs. In ‘Exploratory analyses of results’, we explore this latter point in greater detail by analysing the protein–ligand binding similarities between common PDB training data and this benchmark’s evaluation datasets.

### Exploratory analyses of results

We explore a range of questions to study the common ‘failure’ modes of the baseline methods included in this work, to outline new directions for future research and development efforts in drug discovery.

#### Research question 1

What are the most common types of protein–ligand complexes that all baseline methods fail to predict?

**Extended Data Fig. 2:**
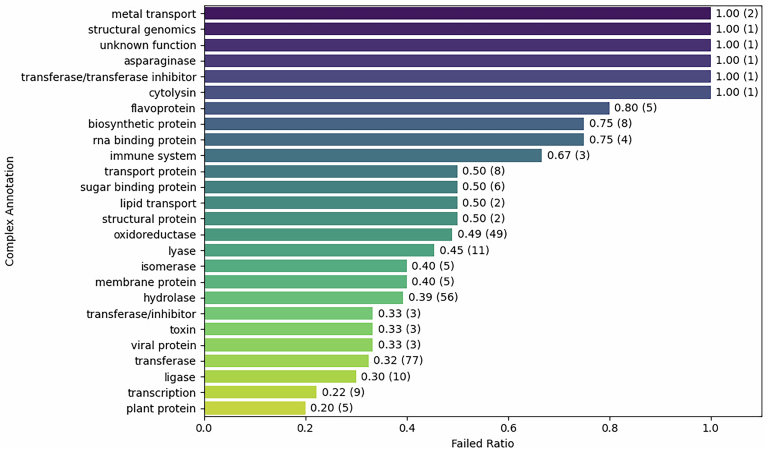
Mispredicted PLI Complex Annotations. Function annotations of the PLI complexes all methods mispredicted (n=129 protein-ligand complexes).

To address this query, we first collected all ligand pose predictions that no method could predict with structural and chemical accuracy (according to the metric r.m.s.d. ≤ 2 Å and PB-Valid). For each of these ‘failed’ ligand poses, we retrieved the PDB’s functional annotation of the protein in complex with this ligand and constructed a histogram to visualize the frequency of these (failed complex) annotations. The results of this analysis are presented in Extended Data Fig. 2, in which we see that metal transport proteins, flavoproteins, biosynthetic proteins, RNA binding proteins, immune system proteins and oxidoreductases are commonly mispredicted by all baseline methods such as Chai-1 and RoseTTAFold-All-Atom (RFAA)^7^, suggesting that these classes of proteins may be largely unaddressed by the most recent DL methods for PLI structure prediction. To illuminate potential future research directions, in the next analysis, we investigate whether this pattern persists specifically for AF3, the most accurate DL cofolding method according to our benchmarking results.

#### Research question 2

What are the most common types of protein–ligand complexes that highly-accurate DL cofolding methods such as AF3 fail to predict?

**Extended Data Fig. 3:**
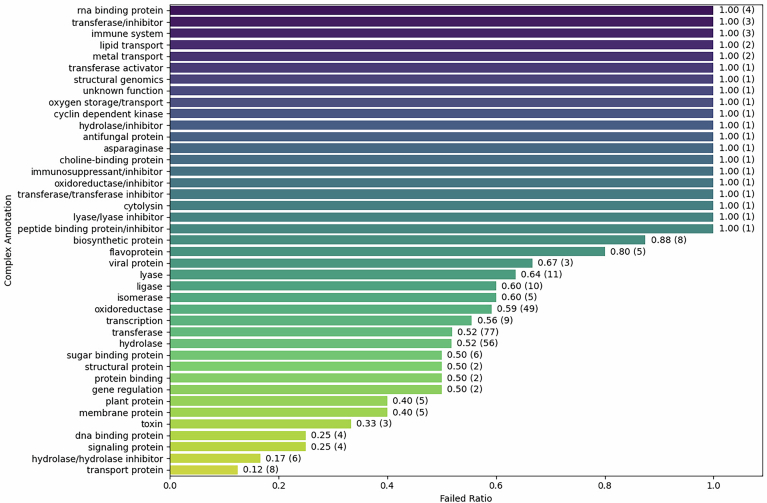
AF3-Mispredicted PLI Complex Annotations. Function annotations of the PLI complexes AF3 mispredicted (n=171 protein-ligand complexes).

For this follow-up question, we linked all of AF3’s failed ligand predictions with corresponding protein function annotations available in the PDB to understand which types of PLI complexes AF3 finds the most difficult to predict (to understand its predictive coverage of important molecular functions). Similarly to the answer to our first research question, Extended Data Fig. 3 shows that, in order of difficulty, AF3 is most challenged to produce ligand poses of high structural and chemical accuracy for ligand-bound RNA binding proteins, immune system proteins, metal transport proteins, biosynthetic proteins, flavoproteins, lyases and oxidoreductases. As several of these classes of proteins have not been well represented in the PDB over the last 50 years (for example, immune system and biosynthetic proteins), in future work, it will be important to ensure that either the performance of new DL methods for PLI structure prediction is expanded to support accurate modelling of these uncommon types of ligand-bound proteins or a broadly applicable fine-tuning method for uncommon types of interactions is proposed.

#### Research question 3

Is lack of protein–ligand binding pose homology to PDB training data (inversely) correlated with the prediction accuracy of each method?

**Extended Data Fig. 4:**
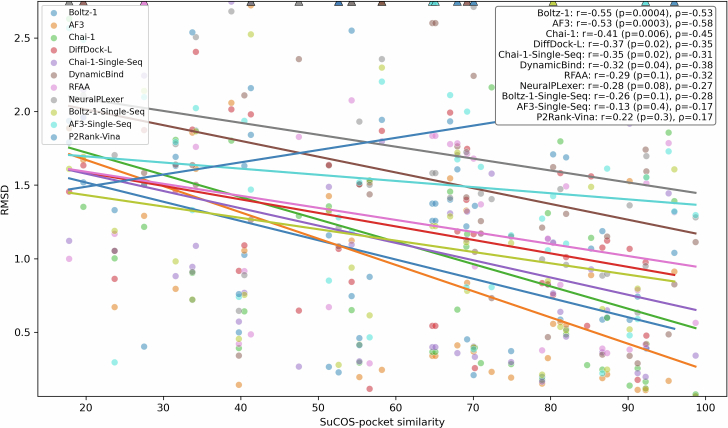
PoseBusters Benchmark Generalization Analysis. Linear regression lines between each method’s PoseBusters Benchmark prediction accuracy (RMSD) and each complex’s training set similarity in terms of SuCOS-pocket similarity (n=41 protein-ligand complexes for each method). Pearson (r, with p-values) and Spearman (*τ*) correlation coefficients were calculated to quantify the relationship between each method’s prediction RMSD and its target’s training set similarity using two-sided tests from SciPy. Reported p-values are unadjusted, as no correction for multiple comparisons was applied.

**Extended Data Fig. 5:**
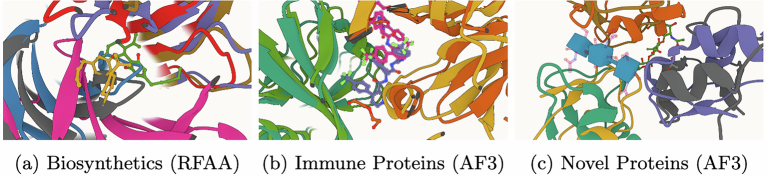
Failure Modes Analysis. Examples of baseline methods’ three failure modes (**a**: biosynthetics, **b**: immune proteins, **c**: novel proteins) discussed in this work.

To understand the impact of protein–ligand binding pose similarity on the performance of each baseline method, we used the PLINDER data resource^36^ to identify (*n* = 41/130 protein–ligand complexes) cluster representatives of the PoseBusters Benchmark dataset based on the product of each complex’s ligand (structure and feature-based) combined overlap score (SuCOS)^48^ and its protein binding pocket’s (structure and sequence-based) similarity^34^, as none of this subset’s prediction targets are contained in any method’s training dataset. For these cluster representatives, with SciPy 1.15.1^49^ we then calculated the Pearson and Spearman correlations (and *P* values) between each method’s complex prediction accuracy (that is, ligand pose r.m.s.d.) and the complex’s maximum SuCOS-binding pocket-based similarity to any complex deposited in the PDB before AF3’s training dataset cutoff of 30 September, 2021. Extended Data Fig. 4 reveals that the performance of all DL methods is correlated with a complex’s similarity to common PDB training data, with (MSA-based) Boltz-1, AF3 and Chai-1 exhibiting the strongest and most statistically significant (*P* < 0.05) correlations. Like concurrent work assessing the performance of cofolding methods in new prediction settings^34^, our findings suggest that although the current generation of DL models (both docking and cofolding methods) for protein–ligand docking and structure prediction can occasionally make accurate predictions for truly new (SuCOS-pocket similarity < 30) protein–ligand complexes, such methods rely (at least in large part) on recapitulating protein–ligand binding patterns seen during training to make accurate predictions for unseen complexes (note that, interestingly, for conventional docking methods such as P2Rank-Vina, this trend is not observed). We conclude our quantitative analyses with an illustration of the different failure modes of each baseline method, as depicted in Extended Data Fig. 5. In this figure, we illustrate that DL methods such as RFAA and AF3 commonly struggle to accurately predict the structure of ligand-binding biosynthetic and immune system proteins, suggesting that these (uncommon) types of PLIs are not well addressed by the current generation of DL-based structure prediction methods, which proposes future research opportunities for interaction-specific modelling (for example, through the use of fine tuning or preference optimization).

## Conclusions

In this Article, we have introduced PoseBench, a unified, broadly applicable benchmark and toolkit for studying the performance of methods for protein–ligand docking and structure prediction. Benchmarking results with PoseBench, summarized in Table 1, suggest that DL cofolding methods generally outperform conventional and DL docking baselines but remain challenged to predict complexes containing new protein–ligand binding poses, with AF3 performing best overall when deep MSAs are available for a target protein (Chai-1 otherwise), regardless of the availability of homologous proteins. Further, we find that several DL methods face difficulties balancing the structural accuracy of their predicted poses with the chemical specificity of their induced protein–ligand interactions, highlighting that future methods may benefit from the introduction of physicochemical loss functions or sampling techniques to bridge this performance gap. Lastly, we observe that some (but not all) DL cofolding methods are highly dependent on the availability of diverse input MSAs to achieve high structural prediction accuracy (for example, AF3 but not Chai-1 or Boltz-1), underscoring the need in future work to elucidate the impact of the availability of MSAs and protein language model embeddings on the training dynamics of biomolecular structure prediction methods. As a publicly available resource, PoseBench is flexible to accommodate new datasets, methods and analyses for protein–ligand docking and structure prediction.

**Table 1:** PoseBench evaluation datasets of protein–(multi)ligand structures

	Astex Diverse	DockGen-E	PoseBusters Benchmark	CASP15
Ligand type	Primary	Primary	Primary	Multi
Source	Ref. ^44^		Ref. ^12^	
Size (total number of ligands)	85	122	130/308	102 (across 19 complexes) → 6 (13) single (multi)ligand complexes
Training data homology	High	Moderate	Low	Low
Top-ranked method (with MSAs)	AF3	AF3	AF3	AF3
Top-ranked method (without MSAs)	Chai-1	Chai-1	Chai-1	NeuralPLexer

## Methods

### PoseBench

The overall goal of PoseBench, our newly designed benchmark for protein–ligand docking and structure prediction, is to provide the research community with a centralized resource with which one can systematically measure, in a variety of macromolecular contexts, the methodological advancements of new conventional and DL methods proposed for this domain. In the following sections, we describe PoseBench’s design and composition (as portrayed in Fig. 1) and how we have used PoseBench to evaluate several recent DL-docking and cofolding methods (as well as a strong conventional baseline algorithm) for protein–ligand structure modelling.

### Benchmark datasets

As shown in Table 1, PoseBench provides users with broadly applicable, preprocessed versions of four datasets with which to evaluate existing or new protein–ligand structure prediction methods: Astex Diverse^44^, PoseBusters Benchmark^12^, and the new DockGen-E and CASP15 PLI datasets that we have manually curated in this work.

#### Astex Diverse dataset

The Astex Diverse dataset is a collection of 85 PLI complexes composed of various drug-like molecules and cofactors known to be of pharmaceutical or agrochemical interest, where a primary (representative) ligand is annotated for each complex. This dataset can be considered an easy benchmarking dataset for methods trained on recent data contained in the PDB in that most of its complexes (deposited in the PDB up to 2007) are known to overlap with the commonly used PDBBind 2020 (time-split) training dataset^14,50^ containing complexes deposited in the PDB before 2019. As such, including this dataset for benchmarking allows one to estimate the breadth of a method’s structure prediction capabilities for important primary ligand–protein complexes represented in the PDB.

To perform unbound (apo) protein–ligand docking with this dataset, we used AF3 to predict the structure of each of its protein complexes, with all ligands and cofactors excluded. We then optimally aligned these predicted protein structures to the corresponding crystal (holo) PLI complex structures using a PLI binding site-focused structural alignment performed using PyMOL^51^, where each binding site is defined as all amino acid residues containing crystallized heavy atoms that are within 10 Å of any crystallized ligand heavy atom. To enable the broad availability of PoseBench’s benchmark datasets in both commercial and academic settings, we also provide unbound (apo) protein structures predicted using the MIT-licensed ESMFold model^45^, although in ‘Results and discussion’ we report results using AF3’s predicted structures as the default data source. We further note that, on average, across all benchmark datasets and methods, AF3’s predicted structures improve the structural accuracy rates of baseline docking methods by 5–10%.

#### PoseBusters Benchmark dataset

Version 2 of the popular PoseBusters Benchmark dataset^12^, which we adopt in this work, contains 308 recent primary ligand–protein complexes deposited in the PDB from 2019 onwards. Accordingly, in contrast with Astex Diverse, this dataset can be considered a moderately difficult benchmark dataset for baseline methods, because many of its complexes do not directly overlap with the most commonly used PDB-based training data. It is important to note that, among all baseline methods, AF3 and Boltz-1 used the most recent PDB training data cutoff of 30 September, 2021, which motivated us to report the results in ‘PoseBusters Benchmark results’ for only the subset of PoseBusters Benchmark complexes (*n* = 130 protein–ligand complexes) deposited in the PDB after this date. Like Astex Diverse, for the PoseBusters Benchmark dataset, we used AF3 (and ESMFold) to predict the apo protein structures of each of its complexes and then performed our PyMOL-based structural binding site alignments.

#### DockGen-E dataset

The original DockGen dataset^13^ contains 189 diverse primary ligand protein complexes, each representing a functionally distinct type of PLI binding pocket according to ECOD domain partitioning^46,47^. Consequently, this dataset can be considered PoseBench’s most difficult primary ligand dataset to model because its PLI binding sites are distinctly uncommon compared with those frequently found in the training datasets of all baseline methods, although it is important to note that these original DockGen complexes were deposited in the PDB from 2019 onward, making this benchmarking dataset partially overlap with the training datasets of baseline DL cofolding methods such as Chai-1, Boltz-1 and AF3. Nonetheless, in line with our initial hypotheses, the benchmarking results in ‘Results and discussion’ demonstrate that no baseline method can adequately predict the PLI binding sites and ligand poses represented by this bespoke subset of the PDB, suggesting that all baseline DL methods have yet to learn broadly applicable representations of protein–ligand binding.

Unfortunately, the original DockGen dataset contains only the primary protein chains representing each new binding pocket after filtering out all non-interacting chains and cofactors in a given biological assembly (bioassembly), which considerably reduces the biophysical context provided to baseline methods to make reasonable predictions. As such, we argue for the need to construct a new dataset that challenges baseline methods (in particular, DL cofolding methods) to predict full bioassemblies containing new PLI binding pockets, which we address with our enhanced version of DockGen called DockGen-E.

To construct DockGen-E, we collected the original DockGen dataset’s PLI binding pocket annotations for each complex. We then retrieved the corresponding first bioassembly listed in the PDB to obtain each PDB entry’s biologically relevant complex, filtering out DockGen complexes for which the first bioassembly could not be mapped to its original PLI binding pocket annotation (which indicates that these original DockGen PLI binding pockets were initially not derived from the PDB’s corresponding first bioassembly). This procedure left 122 biologically relevant assemblies remaining for benchmarking. Like Astex Diverse and PoseBusters Benchmark, for DockGen-E, we then used AF3 (and ESMFold) to predict the unbound (apo) protein structures of each complex in the dataset and structurally aligned the predicted protein structures to their corresponding crystallized PLI binding sites using PyMOL.

#### CASP15 dataset

To assess the multiprimary ligand (that is, multiligand) modelling capabilities of recent methods for protein–ligand docking and structure prediction, with PoseBench, we introduced a preprocessed, DL-ready version of the CASP15 PLI dataset debuted as a first-of-its-kind prediction category in the 15th Critical Assessment of Techniques for Structure Prediction (CASP) competition held in 2022^41^. The CASP15 PLI dataset originally comprised 23 protein–ligand complexes released in the PDB from 2022 onwards, where we subsequently filtered out four complexes based on: (1) whether the CASP organizers ultimately assessed predictions for the complex; and (2) whether they are nucleic acid-ligand complexes with no interacting protein chains. The 19 remaining PLI complexes, which contain a total of 102 (fragment) ligands, consist of a variety of ligand types including single-atom (metal) ions and large drug-sized molecules with up to 92 atoms in each (fragment) ligand. As such, this dataset is appropriate for assessing how well structure prediction methods can model interactions between different (fragment) ligands in the same complex, which can yield insights into the interligand steric clash rates of each method. As with all other benchmark datasets, we used AF3 (and ESMFold) to predict the unbound (apo) structure of each protein complex in the dataset and then performed a PyMOL-based structural alignment of the corresponding PLI binding sites.

#### PLI similarity analysis between datasets

For an investigation of the similarity of PLIs represented in each dataset, in Supplementary Appendix E, we analyse the different types and frequencies of common, ProLIF-annotated protein–ligand binding pocket interactions^52^ natively found within the common PDBBind 2020 training dataset and the Astex Diverse, PoseBusters Benchmark, DockGen-E and CASP15 datasets, respectively, to quantify the diversity of the (predicted) interactions that each dataset can be used to evaluate. In short, we find that the DockGen-E and CASP15 benchmark datasets are the most dissimilar compared with the common PDBBind 2020 training dataset, further illustrating the unique PLI modelling challenges offered by these evaluation datasets.

### Formulated tasks

In this work, we developed PoseBench to focus our analysis on the behaviour of different conventional and DL methods for protein–ligand structure prediction in a variety of macromolecular contexts (for example, with or without inorganic cofactors present). With this goal in mind, below we formalize the structure prediction tasks currently available with PoseBench, with its source code flexibly designed to accommodate new tasks in future work.

#### Primary ligand blind docking

For primary ligand blind docking, each baseline method is provided with a complex’s (multichain) protein sequence and an optional predicted (apo) protein structure as input along with its corresponding (fragment) ligand simplified molecular input line entry system (SMILES) strings, where fragment ligands include the primary binding ligand to be scored as well as all cofactors present in the corresponding crystal structure. In particular, no knowledge of the complex’s PLI binding pocket is provided to evaluate how well each method can: (1) identify the correct PLI binding pockets; and (2) correct ligand poses within each pocket; (3) with high chemical validity; and (4) with specificity for the pockets’ amino acid residues. After all fragment ligands are predicted, PoseBench extracts each method’s prediction of the primary binding ligand and reports evaluation results for these primary predictions.

#### Multiligand blind docking

For multiligand blind docking, each baseline method is provided with a complex’s (multichain) protein sequence and an optional predicted (apo) protein structure as input along with its corresponding (fragment) ligand (SMILES) strings. As in primary ligand blind docking, no knowledge of the PLI binding pockets is provided, which offers the opportunity to evaluate not only PLI binding pocket and conformation prediction accuracy but, in the context of multibinding ligands, also interligand steric clash rates.

### Metrics

#### Traditional metrics

For PoseBench, we reference two key metrics in the field of structural bioinformatics: the r.m.s.d. and local distance difference test (lDDT)^53^. The r.m.s.d. between a predicted three-dimensional conformation (with atomic positions ${\hat{x}}_{i}$ for each of the molecule’s *n* heavy atoms) and the ground-truth (crystal structure) conformation (*x*_*i*_) is defined as

1 $$\,\mathrm{r.m.s.d.}\,=\sqrt{\frac{1}{n}\mathop{\sum }\limits_{i=1}^{n}{\parallel {\hat{x}}_{i}-{x}_{i}\parallel }^{2}}.$$

The lDDT score, which is commonly used to compare predicted and ground-truth protein three-dimensional structures, is defined as

2 $$\,\mathrm{lDDT}\,=\frac{1}{N}\mathop{\sum }\limits_{i=1}^{N}\frac{1}{4}\mathop{\sum }\limits_{k=1}^{4}\left(\frac{1}{| {{\mathcal{N}}}_{i}| }\sum _{j\in {{\mathcal{N}}}_{i}}\varTheta (| {\hat{d}}_{ij}-{d}_{ij}| < {\Delta }_{k})\right),$$

where *N* is the total number of heavy atoms in the ground-truth structure; ${{\mathcal{N}}}_{i}$ is the set of neighbouring atoms of atom *i* within the inclusion radius *R*_*o*_ = 15 Å in the ground-truth structure, excluding atoms from the same residue; ${\hat{d}}_{ij}$ (*d*_*ij*_) is the distance between atoms *i* and *j* in the predicted (ground-truth) structure; Δ_*k*_ are the distance tolerance thresholds (that is, 0.5 Å, 1 Å, 2 Å and 4 Å); *Θ*(*x*) is a step function that equals 1 if *x* is true, and 0 otherwise; and $| {{\mathcal{N}}}_{i}|$ is the number of neighbouring atoms for atom *i*. As originally proposed in ref. ^41^, in this study, we adopted the PLI-specific variant of lDDT for scoring multiligand complexes, which calculates lDDT scores to compare predicted and ground-truth protein–(multi)ligand complex structures following optimal (chain-wise and residue-wise) structural alignment of the predicted and ground-truth PLI binding pockets.

Lastly, we also measure the molecule validity rates of each predicted PLI complex pose using the PoseBusters software suite (that is, PB-Valid)^12^. This suite runs several important chemical and structural sanity checks for each predicted pose, including energy ratio inspection and geometric (for example, flat aliphatic ring) assertions, which provide a secondary filter of accurate poses that are also chemically and structurally meaningful.

#### New metrics

The r.m.s.d., lDDT and PB-Valid metrics of a protein–ligand binding structure provide useful characterizations of how accurate and reasonable a predicted pose is. However, a key limitation of these metrics is that they do not measure how well a predicted pose resembles a native pose when comparing their induced PLIFs. Recently, in ref. ^37^, a complementary benchmarking metric, PLIF-valid, was introduced which assesses DL methods’ recovery rates of known PLIs. However, this metric only reports a strict recall rate of each method’s interaction types rather than a continuous measure of how well each method’s interactions match the distribution of crystallized PLIs. Moreover, in drug discovery, a primary concern when designing new drug candidates is ensuring that they produce amino acid-specific types of interactions (and not others); hence, we desire each baseline method to recall the correct types of PLIs for each pose and to avoid predicting (that is, hallucinating) types of interactions that are not natively present. Consequently, we argue that an ideal PLI-aware benchmarking metric is a single continuous metric that assesses the recall and precision of a method’s predicted distribution of amino acid-specific PLIFs. To this end, we propose two new benchmarking metrics, PLIF-EMD and PLIF-WM.

For each PLI complex, PLIF-EMD measures the earth mover’s distance (EMD)^54^ between a method’s predicted histogram of PLI type counts **u** (specific to each type of interaction) and the corresponding native histogram **v**, where each histogram of interaction type counts is represented as a one-dimensional discrete distribution. Formally, this equates to computing the Wasserstein distance between these two one-dimensional distributions **u** and **v** as

3 $$\,\mathrm{PLIF-EMD}\,\,:= \,{l}_{1}(\mathbf{u},\mathbf{v})=\mathop{\inf }\limits_{\uppi \in {\rm{\Pi }}(\mathbf{u},\mathbf{v})}{\int}_{{\mathbb{R}}\times {\mathbb{R}}}| x-y| {\rm{d}}\uppi (x,y),$$

where Π(**u**, **v**) denotes the set of distributions on ${\mathbb{R}}\times {\mathbb{R}}$ whose marginals, **u** and **v**, are on the first and second factors, respectively. To penalize a baseline method for producing non-native interaction types, we unify the bins in each histogram before converting them into one-dimensional discrete representations. Namely, to perform this calculation, each PLI is first represented as a fingerprint tuple of < ligand type, amino acid type, interaction type > as determined by the software tool ProLIF^52^ and then grouped to count each type of tuple to form a histogram. As such, a lower PLIF-EMD value implies a better continuous agreement between predicted and native interaction histograms. PLIF-WM, derived from PLIF-EMD, assesses the WM score of a pair of PLIF histograms. Specifically, to obtain a more benchmarking-friendly score ranging from 0 to 1 (higher is better), we define PLIF-WM as

4 $$\,\mathrm{PLIF-WM}\,\,:= \,1-\frac{\,\mathrm{PLIF-EMD}-{\mathrm{PLIF-EMD}}_{{\rm{min}}}}{{\mathrm{PLIF-EMD}}_{{\rm{max}}}-{\mathrm{PLIF-EMD}}_{{\rm{min}}}},$$

where PLIF-EMD_min_ and PLIF-EMD_max_ denote the minimum (best) and maximum (worst) values of PLIF-EMD, respectively. As a metric normalized relative to each collection of the latest baseline methods, PLIF-WM allows one to quickly identify which of the latest methods has the greatest capacity to produce realistic distributions of PLIs. As a practical note, we use SciPy 1.15.1^49^ to provide users of PoseBench with an optimized implementation of PLIF-EMD and thereby PLIF-WM.

### Baseline methods and experimental set-up

#### Overview

We designed PoseBench to answer specific modelling questions for PLI complexes such as: (1) which types of methods (if any) can predict both common and uncommon PLI complexes with high structural and chemical accuracy; and (2) which most accurately predict multiligand structures without steric clashes? In the following sections, we discuss which types of methods we evaluate in our benchmark and how we evaluate each method’s predictions for PLI complex targets.

#### Method categories

As illustrated in Fig. 1, to explore a range of the most well-known or recent methods to date, we divide PoseBench’s baseline methods into one of three categories: (1) conventional algorithms; (2) DL docking algorithms; and (3) DL cofolding algorithms.

As a representative algorithm for conventional protein–ligand docking, we pair AutoDock Vina (v.1.2.5)^55^ for molecular docking with P2Rank for protein–ligand binding site prediction^56^ to form a strong conventional (blind) docking baseline (P2Rank-Vina) for comparison with DL methods. To represent DL docking methods, we include DiffDock-L^13^ for docking with static protein structures and DynamicBind^9^ for flexible docking. Lastly, to represent some of the latest DL cofolding methods, we include NeuralPLexer^10^, RFAA^7^, Chai-1^30^, Boltz-1^31^ (versus Boltz-2^33^ for the sake of time-split benchmarking validity) and AF3^11^. For interested readers, each method’s input and output data formats are described in Supplementary Appendix F.

#### Prediction and evaluation procedures

The PLI complex structures that each method predicts are subsequently evaluated using different structural and chemical accuracy and molecule validity metrics depending on whether the targets are primary or multiligand complexes. In ‘Metrics’, we provide formal definitions of PoseBench’s evaluation metrics. Note that if a method’s prediction raises any errors in subsequent scoring stages (for example, due to missing entities or formatting violations), the prediction is excluded from the evaluation.

#### Primary ligand evaluation

For primary ligand targets, we report each method’s percentage of (top-1) ligand conformations within 2 Å of the corresponding crystal ligand structure (r.m.s.d. ≤ 2 Å), using 1 Å to instead assess whether the predicted ligand’s heavy atom centroid (that is, binding pocket) was correct (c.r.m.s.d. ≤ 1 Å), as well as the percentage of such ‘correct’ ligand conformations that are also considered to be chemically and structurally valid according to the PoseBusters software suite^12^ (r.m.s.d. ≤ 2 Å and PB-Valid). Importantly, as described in ‘Metrics’, we also report each method’s new PLIF-WM scores to study the relationship between its structural accuracy and chemical specificity.

#### Multiligand evaluation

Similarly to the protein–ligand scoring procedure employed in the CASP15 competition^41^, for multiligand targets, we report each method’s (top-1) percentage of ‘correct’ (binding site-superimposed) ligand conformations (r.m.s.d. ≤ 2 Å) as well as violin plots of the r.m.s.d. and PLI-specific lDDT scores of its protein–ligand conformations across all (fragment) ligands within the benchmark’s multiligand complexes (see Supplementary Appendix G for these plots). Notably, this latter metric, referred to as lDDT-PLI, allows one to evaluate specifically how well each method can model protein–ligand structural interfaces. In addition, we report each method’s PB-Valid rates (calculated once for each multiligand complex) and PLIF-WM scores.

### Reporting summary

Further information on research design is available in the Nature Portfolio Reporting Summary linked to this article.

## Supplementary information

Supplementary Information Appendices A–G and Supplementary Figs. 1–27.

Reporting Summary

## Data Availability

The PoseBench datasets and benchmark results are available via Zenodo at 10.5281/zenodo.17536252 (ref. ^57^) under a Creative Commons Attribution 4.0 International Public License, with further licensing discussed in Supplementary Appendix A and detailed dataset documentation (for example, of AF3’s predicted protein structure accuracy) provided in Supplementary Appendix D.

## Code availability

The PoseBench codebase, documentation and tutorial notebooks are available via GitHub at https://github.com/BioinfoMachineLearning/PoseBench and via Zenodo at 10.5281/zenodo.17536423 (ref. ^58^) under a permissive MIT license, with further licensing and broader impacts discussed in Supplementary Appendices A and B. In particular, the code makes use of the Python packages hydra-core 1.3.2, biopandas 0.5.1.dev0, biopython 1.79, meeko 0.6.0a3, numpy 1.26.4, pandas 1.5.0, posebusters 0.4.5, posecheck 1.1, prolif 2.0.3, pypdb 2.3, rdkit 2025.3.5, scikit-learn 1.1.2, seaborn 0.12.2 and spyrmsd 0.5.2.
