# Supplementary material for: Assessing the potential of deep learning for protein–ligand docking
Source: Nat Mach Intell. 2025 Dec 31;8(1):32–41. doi: 10.1038/s42256-025-01160-1 (PMC12851923; doi:10.1038/s42256-025-01160-1)
Supplement: Supplementary file 1 — Appendices A–G and Supplementary Figs. 1–27. [file 42256_2025_1160_MOESM1_ESM.pdf]

nature machine intelligence

Supplementary information

https://doi.org/10.1038/s42256-025-01160-1

Assessing the potential of deep learning for protein–ligand docking

In the format provided by the authors and unedited

Appendices

A Availability **2**

B Broader impacts **2**

C Compute resources **2**

D Documentation for datasets **3**
D.1 Astex Diverse Set - Primary Ligand Docking (Difficulty: *Easy*) 6
D.2 PoseBusters Benchmark Set - Primary Ligand Docking (Difficulty: *Intermediate*) 7
D.3 DockGen-E Set - Primary Ligand Docking (Difficulty: *Challenging*) 8
D.4 CASP15 Set - Multi-Ligand Docking (Difficulty: *Challenging*) 10

E Analysis of protein-ligand interactions **12**
E.1 Dataset protein-ligand interaction distributions 12
E.2 Baseline method protein-ligand interaction distributions 13

F Additional method descriptions **16**
F.1 Input and output formats 16

G Additional results **19**
G.1 Expanded primary ligand results 19
G.1.1 Primary ligand RMSD results 19
G.2 Expanded CASP15 results 21
G.2.1 Overview of expanded results 21
G.2.2 Multi-ligand RMSD and lDDT-PLI 21
G.2.3 All single-ligand results 21
G.2.4 Single and multi-ligand results for *public* targets 21

Appendix A Availability

The PoseBench codebase and tutorial notebooks are available under an MIT license at https://github.com/BioinfoMachineLearning/PoseBench. Preprocessed datasets and benchmark method predictions and results are available on Zenodo [1] under a CC-BY 4.0 license, of which the Astex Diverse and PoseBusters Benchmark datasets [2] and the DockGen-E dataset are associated with a CC-BY 4.0 license, and of which the CASP15 dataset [3], as a mixture of publicly and privately available resources, is partially licensed. In particular, 15 (4 single-ligand and 11 multi-ligand targets) of the 19 CASP15 protein-ligand interaction (PLI) complexes evaluated with PoseBench are publicly available, whereas the remaining 4 (2 single-ligand and 2 multi-ligand targets) are confidential and, for the purposes of future benchmarking and reproducibility, must be requested directly from the CASP organizers. Notably, the pre-holo-aligned protein structures predicted by AlphaFold 3 (AF3) for these four benchmark datasets (available on Zenodo [1]) must only be used in accordance with AF3's Terms of Use, whereas the pre-holo-aligned protein structures predicted by ESMFold for these four benchmark datasets (available on Zenodo [1]) are available under a permissive MIT license. Lastly, our use of the PoseBusters software suite for molecule validity checking is permitted under a BSD-3-Clause license.

Appendix B Broader impacts

Our benchmark unifies protein-ligand structure prediction datasets, methods, and tasks to enable enhanced insights into the real-world utility of such methods for accelerated drug discovery and energy research. We acknowledge the risk that, in the hands of "bad actors", such technologies may be used with harmful ends in mind. However, it is our hope that efforts in elucidating the performance of recent protein-ligand structure prediction methods in various macromolecular contexts will disproportionately influence the positive societal outcomes of such research such as improved medicines and subsequent clinical outcomes as opposed to possible negative consequences such as the development of new bioweapons.

Appendix C Compute resources

To produce the results presented in this work, we ran a high performance computing sweep that concurrently utilized 12 80GB NVIDIA A100 GPUs for 14 days in total to run inference with each baseline method three times (where applicable), where each baseline deep learning (DL) method required approximately 24 hours of GPU compute to complete its inference runs (except for multiple sequence alignment (MSA)-dependent AF3 and RoseTTAFold-All-Atom (RFAA), which respectively took approximately 4 weeks and 2 weeks to finish their inference runs for each benchmark dataset). Notably, due to RFAA and AF3's significant storage requirements for running inference with their MSA databases, we utilized approximately 6 TB of solid-state storage space in total to benchmark all baseline methods. Lastly, in terms of CPU requirements, our experiments utilized approximately 64 concurrent CPU threads for AutoDock Vina inference (as an upper bound) and 60 GB of CPU RAM. Note that

Table C1: The average runtime (in seconds) and peak memory usage (in GB) of each baseline method on a 25% subset of the Astex Diverse dataset (using an NVIDIA 80GB A100 GPU for benchmarking). The symbol - denotes a result that could not be estimated. Where applicable, an integer enclosed in parentheses indicates the number of samples drawn from a particular baseline method.

Method	Runtime (s)	CPU Memory Usage (GB)	GPU Memory Usage (GB)
P2Rank-Vina (40)	1,283.70	9.62	0.00
DiffDock-L (5)	88.33	8.99	70.42
DynamicBind (5)	146.99	5.26	18.91
NeuralPLexer (5)	29.10	11.19	31.00
RoseTTAFold-All-Atom (1)	3,443.63	55.75	72.79
Chai-1 (5)	114.86	58.49	56.21
Boltz-1 (5)	173.35	37.62	31.41
AF3 (5)	3,049.41	-	-

an additional 4-5 weeks of compute were spent performing initial (non-sweep) versions of each experiment during PoseBench's initial phase of development.

As a more formal investigation of the computational resources required to run each baseline method in this work, in Table C1 we list the average runtime (in seconds) and peak CPU (GPU) memory usage (in GB) consumed by each method when running them on a 25% subset of the Astex Diverse dataset. We find that NeuralPLexer provides the lowest computational runtime and DynamicBind the lowest peak CPU and GPU memory requirements during benchmarking.

Appendix D Documentation for datasets

Below, we provide detailed documentation for each dataset included in our benchmark, summarized in Table 1 of the main text. Each dataset is freely available for download from the benchmark's accompanying Zenodo data record [1] under a CC-BY 4.0 license. In lieu of being able to create associated metadata for each of our macromolecular datasets using an ML-focused library such as Croissant [4] (due to file type compatibility issues), instead, we report structured metadata for our preprocessed datasets using Zenodo's web user interface [1]. Note that, for all datasets, we authors bear all responsibility in case of any violation of rights regarding the usage of such datasets.

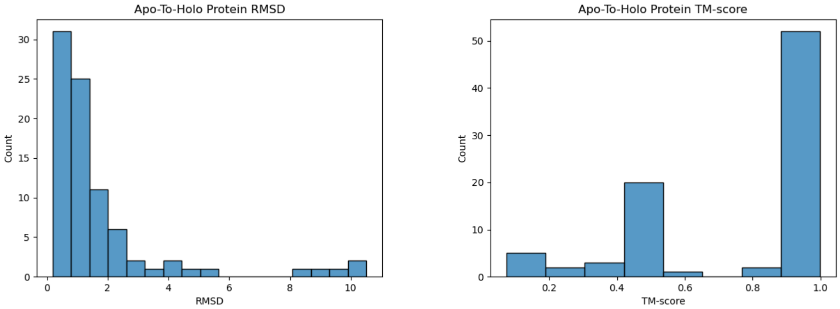

(a) RMSD of AF3's predictions.   (b) TM-score of AF3's predictions.

Fig. D1: Accuracy of AF3's predicted protein structures for the Astex Diverse dataset.

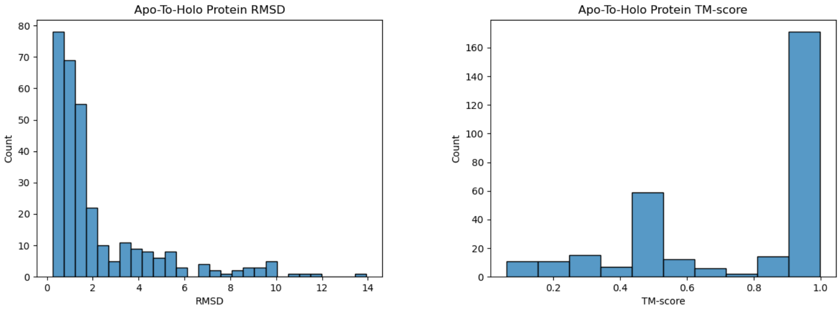

(a) RMSD of AF3's predictions.   (b) TM-score of AF3's predictions.

Fig. D2: Accuracy of AF3's predicted protein structures for the PoseBusters Benchmark dataset.

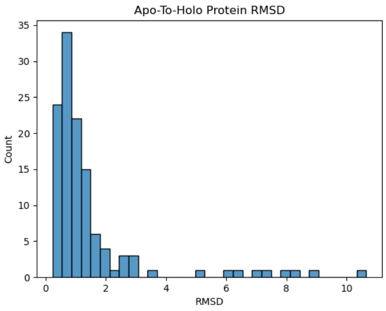

(a) RMSD of AF3's predictions.

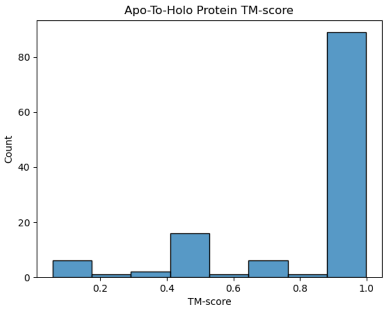

(b) TM-score of AF3's predictions.

Fig. D3: Accuracy of AF3's predicted protein structures for the DockGen dataset.

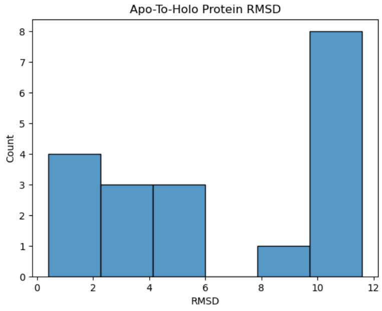

(a) RMSD of AF3's predictions.

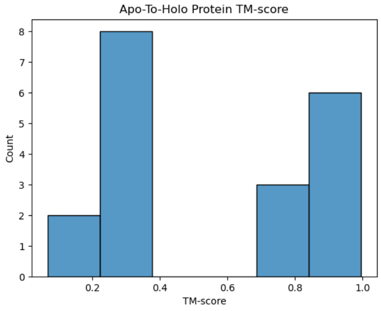

(b) TM-score of AF3's predictions.

Fig. D4: Accuracy of AF3's predicted protein structures for the CASP15 dataset.

D.1 Astex Diverse Set - Primary Ligand Docking (Difficulty: *Easy*)

A common drug discovery task is to screen several novel drug-like molecules against a target protein in rapid succession. The Astex Diverse dataset was originally developed with this application in mind, as it features many therapeutically relevant 3D molecules for computational modeling.

- **Motivation** Several downstream drug discovery efforts rely on having access to high-quality molecular data for docking.
- **Collection** For this dataset, which was originally compiled by Hartshorn et al. [5], we adopt the version further prepared by Buttenschoen et al. [2].
- **Composition** The dataset consists of 85 primary ligand protein complexes deposited in the PDB up to 2007. As such, this dataset can be considered an easy benchmarking dataset since many of its complexes may be found in DL methods' PDB-based training datasets. For each of these complexes, we obtained high-accuracy predicted protein structures using AF3. The accuracy of the AF3-predicted structures is measured in terms of their RMSD and TM-score [6] compared to the corresponding crystal protein structures and is visualized in Figure D1. Notably, after alignment with the crystalized (holo) PLI binding pocket residues, 63.53% (54.12% with ESMFold) of the predicted structures have a global RMSD below 4 Å and TM-score above 0.7, indicating that most of the dataset's proteins have a reasonably accurate predicted structure.
- **Hosting** Our preprocessed version of the dataset (https://doi.org/10.5281/zenodo.17536252) can be downloaded from the benchmark's Zenodo data record at https://zenodo.org/records/17536252/files/astex_diverse_set.tar.gz.
- **Licensing** We have released our preprocessed version of the dataset under a CC-BY 4.0 license. The original PoseBusters Benchmark dataset is available under a CC-BY 4.0 license on Zenodo [7]. The pre-holo-aligned protein structures predicted by AF3 for this dataset (available on Zenodo [1]) must only be used in accordance with AF3's Terms of Use.
- **Maintenance** We will announce any errata discovered in or changes made to the dataset using the benchmark's GitHub repository at https://github.com/BioinfoMachineLearning/PoseBench.
- **Uses** This dataset of predicted (apo) and crystal (holo) protein PDB and crystal (holo) ligand SDF files can be used for primary ligand docking or protein-ligand structure prediction.
- **Metrics** Ligand Centroid RMSD $\leq$ 1 Å, Ligand Pose RMSD $\leq$ 2 Å, PoseBusters-Valid (PB-Valid), and PLIF-WM.

D.2 PoseBusters Benchmark Set - Primary Ligand Docking (Difficulty: *Intermediate*)

Like the Astex Diverse dataset, the PoseBusters Benchmark dataset was originally developed for docking individual ligands to target proteins. However, this dataset features a larger and more challenging collection of PLI complexes for computational modeling.

- **Motivation** Data sources of challenging primary ligand protein complexes for molecular docking are critical for the development of future docking methods.
- **Collection** For this dataset, we adopt the version introduced by Buttenschoen et al. [2].
- **Composition** The dataset consists of 308 primary ligand protein complexes deposited in the PDB in 2019 and after. As such, this dataset poses a moderate challenge for DL methods, since several of such methods were trained on data deposited before this cutoff date (notably except for Chai-1 and AF3/Boltz-1 which used training cutoff dates of January 12, 2021 and September 30, 2021, respectively). For each of the dataset's complexes, we obtained high-accuracy predicted protein structures using AF3. The accuracy of the AF3-predicted structures is measured in terms of their RMSD and TM-score compared to the corresponding crystal protein structures and is visualized in Figure D2. Notably, after alignment with the crystalized (holo) PLI binding pocket residues, 59.09% (53.25% with ESMFold) of the predicted structures have a global RMSD below 4 Å and TM-score above 0.7, indicating that most of the dataset's proteins have a reasonably accurate predicted structure.
- **Hosting** Our preprocessed version of the dataset (https://doi.org/10.5281/zenodo.17536252) can be downloaded from the benchmark's Zenodo data record at https://zenodo.org/records/17536252/files/posebusters_benchmark_set.tar.gz.
- **Licensing** We have released our preprocessed version of the dataset under a CC-BY 4.0 license. The original dataset is available under a CC-BY 4.0 license on Zenodo [7]. The pre-holo-aligned protein structures predicted by AF3 for this dataset (available on Zenodo [1]) must only be used in accordance with AF3's Terms of Use.
- **Maintenance** We will announce any errata discovered in or changes made to the dataset using the benchmark's GitHub repository at https://github.com/BioinfoMachineLearning/PoseBench.
- **Uses** This dataset of predicted (apo) and crystal (holo) protein PDB and crystal (holo) ligand SDF files can be used for primary ligand docking or protein-ligand structure prediction.
- **Metrics** Ligand Centroid RMSD $\leq$ 1 Å, Ligand Pose RMSD $\leq$ 2 Å, PoseBusters-Valid (PB-Valid), and PLIF-WM.

D.3 DockGen-E Set - Primary Ligand Docking (Difficulty: *Challenging*)

The DockGen dataset was originally designed for binding individual ligands to target proteins within functionally novel PLI binding pockets [8], filtering out any protein chains not associated with a novel pocket, which can remove important biomolecular context for DL methods to make their predictions. In this work, we introduced DockGen-E, an enhanced version of DockGen that has each method predict the full biologically relevant assembly of each novel pocket to expand their structural prediction contexts (n.b., which is specifically important to achieve best performance with DL co-folding methods such as AF3). As such, this new dataset is useful for evaluating how well each baseline method can predict complexes containing functionally distinct binding pockets compared to those on which the method may have *primarily* been trained.

- **Motivation** Data sources of PLI complexes representing novel primary ligand binding pockets are critical for the development of generalizable docking methods.
- **Collection** To curate this dataset, we collected the original dataset's protein and ligand binding pocket annotations for each complex introduced by Corso et al. [8]. Subsequently, we retrieved the corresponding first biological assembly listed in the PDB to obtain each PDB entry's biologically relevant complex, filtering out complexes for which the first assembly could not be mapped to its original protein and ligand binding pocket annotation. This procedure left 122 biologically relevant assemblies remaining for benchmarking. Important to note is that these original DockGen complexes were deposited in the PDB from 2019 onward, making this benchmarking dataset partially overlap with the training datasets of multiple DL co-folding baseline methods such as NeuralPLexer, Chai-1, Boltz-1, and AF3. Nonetheless, our benchmarking results in the main text demonstrate that baseline DL methods are challenged to find the correct (novel) binding pocket conformations represented by this dataset, suggesting that all baseline DL models have yet to learn truly comprehensive representations of protein-ligand binding.
- **Composition** The dataset consists of 122 primary ligand protein complexes, for each of which we obtained high-accuracy predicted protein structures using AF3. The accuracy of the AF3-predicted structures is measured in terms of their RMSD and TM-score compared to the corresponding crystal protein structures and is visualized in Figure D3. Notably, after alignment with the crystalized (holo) PLI binding pocket residues, 74.59% (57.38% with ESMFold) of the predicted structures have a global RMSD below 4 Å and TM-score above 0.7, indicating that most of the dataset's proteins have a reasonably accurate predicted structure.
- **Hosting** Our preprocessed version of the dataset (https://doi.org/10.5281/zenodo.17536252) can be downloaded from the benchmark's Zenodo data record at https://zenodo.org/records/17536252/files/dockgen_set.tar.gz.
- **Licensing** We have released our preprocessed version of the DockGen-E dataset under a CC-BY 4.0 license. The original DockGen dataset is available under an MIT license on Zenodo [9], and the DockGen-E dataset along with its pre-holo-aligned protein structures predicted by AF3 is also available on Zenodo [1].

Notably, these AF3-predicted protein structures must only be used in accordance with AF3's Terms of Use.

- **Maintenance** We will announce any errata discovered in or changes made to the dataset using the benchmark's GitHub repository at https://github.com/BioinfoMachineLearning/PoseBench.
- **Uses** This dataset of predicted (apo) and crystal (holo) protein PDB and crystal (holo) ligand PDB files can be used for primary ligand docking or protein-ligand structure prediction.
- **Metrics** Ligand Centroid RMSD $\leq$ 1 Å, Ligand Pose RMSD $\leq$ 2 Å, PoseBusters-Valid (PB-Valid), and PLIF-WM.

D.4 CASP15 Set - Multi-Ligand Docking (Difficulty: *Challenging*)

As the most distinct of our benchmark's four evaluation datasets, the CASP15 PLI dataset was created to represent the new protein-ligand modeling category in the 15th Critical Assessment of Techniques for Structure Prediction (CASP) competition. Whereas datasets such as PoseBusters Benchmark and Astex Diverse feature solely primary ligand protein complexes, the CASP15 dataset provides the research community with a variety of challenging organic (e.g., drug molecules) and inorganic (e..g., ion) cofactors for *multi*-ligand biomolecular modeling and scoring.

- **Motivation** Multi-ligand evaluation datasets for molecular docking provide the rare opportunity to assess how well baseline methods can model intricate PLIs while avoiding troublesome inter-ligand steric clashes. Additionally, accurate modeling of multi-ligand complexes in future work may lead to improved algorithms for computational enzyme design and regulation [10].
- **Collection** For this dataset, we manually collect each publicly and privately available CASP15 protein-bound ligand complex structure compatible with protein-ligand (e.g., non-nucleic acid) benchmarking.
- **Composition** The dataset consists of 102 (86) fragment ligands contained within 19 (15) separate (publicly available) protein complexes, of which 6 (2) and 13 (2) of these complexes are single and multi-ligand complexes, respectively. Importantly, each of such complexes (if publicly available) was released in the PDB after 2022, making this benchmarking dataset strictly non-overlapping with the training datasets of all baseline methods. The accuracy of the dataset's AF3-predicted structures is measured in terms of their RMSD and TM-score compared to the corresponding crystal protein structures and is visualized in Figure D4. Notably, after alignment with the crystalized (holo) PLI binding pocket residues, 36.84% and 20.00% (26.32% and 13.33% with ESMFold) of the total and publicly available predicted structures, respectively, have a global RMSD below 4 Å and TM-score above 0.7, indicating that a portion of the dataset's proteins has a reasonably accurate predicted structure. Given the much larger structural assemblies of this dataset's protein complexes compared to those of the other benchmark datasets, we believe the accuracy of these predictions may be improved with advancements in machine learning modeling of biomolecular assemblies.
- **Hosting** Our preprocessed version of (the publicly available version of) this dataset (https://doi.org/10.5281/zenodo.17536252) can be downloaded from the benchmark's Zenodo data record at https://zenodo.org/records/17536252/files/casp15_set.tar.gz.
- **Licensing** We have released our preprocessed version of the (public) dataset under a CC-BY 4.0 license. The original (public) dataset is free for download via the RCSB PDB [11], whereas the dataset's remaining (private) complexes must be manually requested from the CASP organizers. The pre-holo-aligned protein structures predicted by AF3 for this dataset (available on Zenodo [1]) must only be used in accordance with AF3's Terms of Use.

- **Maintenance** We will announce any errata discovered in or changes made to the dataset using the benchmark's GitHub repository at https://github.com/BioinfoMachineLearning/PoseBench.
- **Uses** This dataset of predicted (apo) and crystal (holo) protein PDB and crystal (holo) ligand PDB files can be used for multi-ligand docking or protein-ligand structure prediction.
- **Metrics** (Fragment) Ligand Pose RMSD $\leq$ 2 Å, (Complex) PoseBusters-Valid (PB-Valid), and (Complex) PLIF-WM.

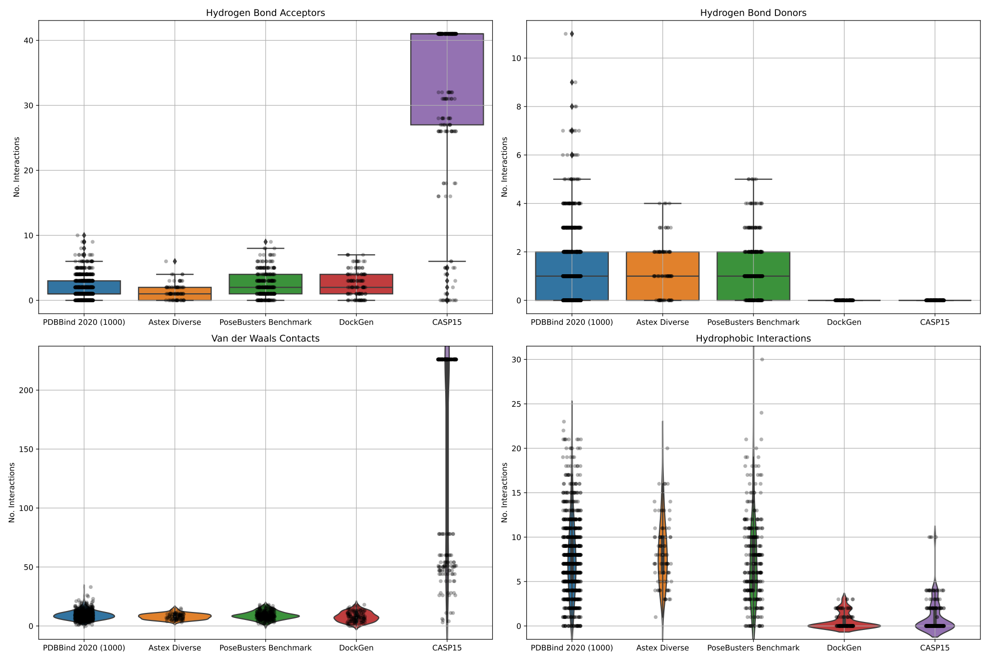

Fig. E5: Comparative analysis of evaluation dataset protein-ligand interactions.

Appendix E Analysis of protein-ligand interactions

E.1 Dataset protein-ligand interaction distributions

Inspired by a similar analysis presented in the PoseCheck benchmark [12], in this section, we study the frequency of different types of protein-ligand (pocket-level) interactions such as van der Waals contacts and hydrophobic interactions occurring natively within (n.b., a size-1000 random subset of) the commonly-used PDBBind 2020 docking training dataset (i.e., PDBBind 2020 (1000)) as well as the Astex Diverse, PoseBusters Benchmark, DockGen, and CASP15 benchmark datasets, respectively. In particular, these measures allow us to better understand the diversity of interactions each baseline method within the PoseBench benchmark is tasked to model, within the context of each evaluation dataset. Furthermore, these measures directly indicate which benchmark datasets are most *dissimilar* from commonly used training data for baseline methods. Figure E5 displays the results of this analysis.

Overall, we find that the PDBBind 2020, Astex Diverse, and PoseBusters Benchmark datasets contain similar types and frequencies of interactions, with the PoseBusters Benchmark dataset containing slightly more hydrogen bond acceptors (∼3 vs 1) and fewer van der Waals contacts (∼5 vs 8) on average compared to the PDBBind 2020 dataset. However, we observe a more notable difference in interaction types and frequencies between the DockGen and CASP15 datasets and the three other

datasets. Specifically, we find these two benchmark datasets contain a notably different quantity of hydrogen bond acceptors and donors (n.b., ∼40 for CASP15), van der Waals contacts (∼200 for CASP15), and hydrophobic interactions (∼2 for DockGen) on average. These dataset-level interaction disparities may partially explain the baseline-challenging DockGen benchmarking results reported in Section 2 of the main text.

Also particularly interesting to note is the CASP15 dataset's bimodal distribution of van der Waals contacts, suggesting that the dataset contains two primary classes of interacting ligands giving rise to van der Waals interactions. One possible explanation for this phenomenon is that the CASP15 prediction targets, in contrast to the PDBBind, Astex Diverse, PoseBusters Benchmark, and DockGen targets, consist of a variety of both organic (e.g., drug-like molecules) and inorganic (e.g., metal) cofactors.

E.2 Baseline method protein-ligand interaction distributions

Intrigued by the dataset interaction patterns presented in Figure E5, here we further investigate the predicted PLIs produced by each baseline method for each evaluation dataset to study which DL methods can most faithfully reproduce the native distribution of PLIs within each dataset. Our results in Figures E6, E7, E8, and E9 suggest that AF3 demonstrates the best overall ability to recapitulate the crystalized PLIs observed within these datasets, in line with the PLIF-WM benchmarking results presented in Section 2 of the main text. Nonetheless, its predicted interaction distributions, in particular for the DockGen and CASP15 datasets, have much room for improvement, especially for more structured interactions such as hydrogen bonds.

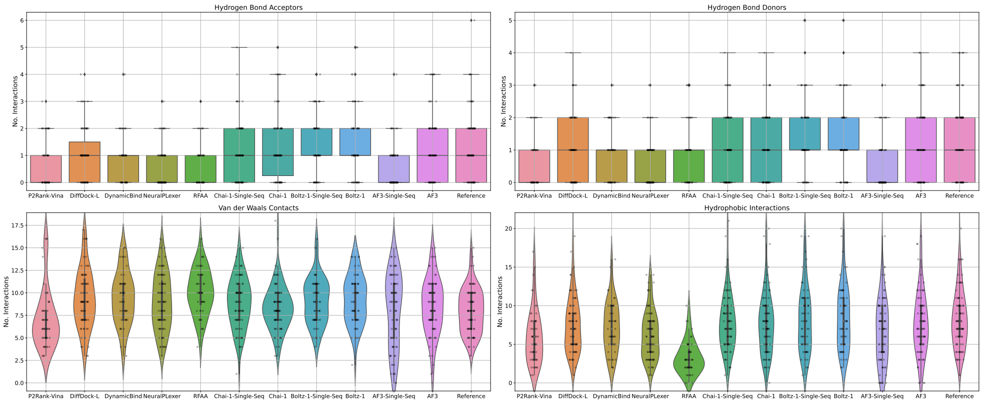

Fig. E6: Comparative analysis of Astex Diverse protein-ligand interactions.

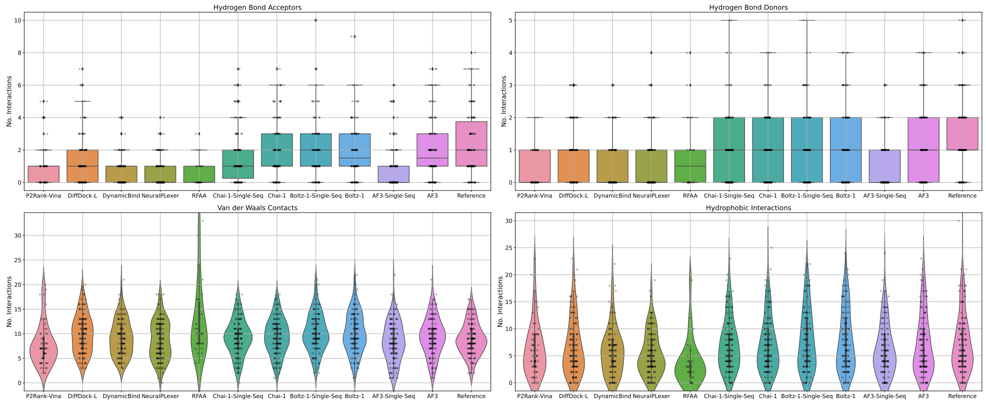

Fig. E7: Comparative analysis of PoseBusters Benchmark protein-ligand interactions.

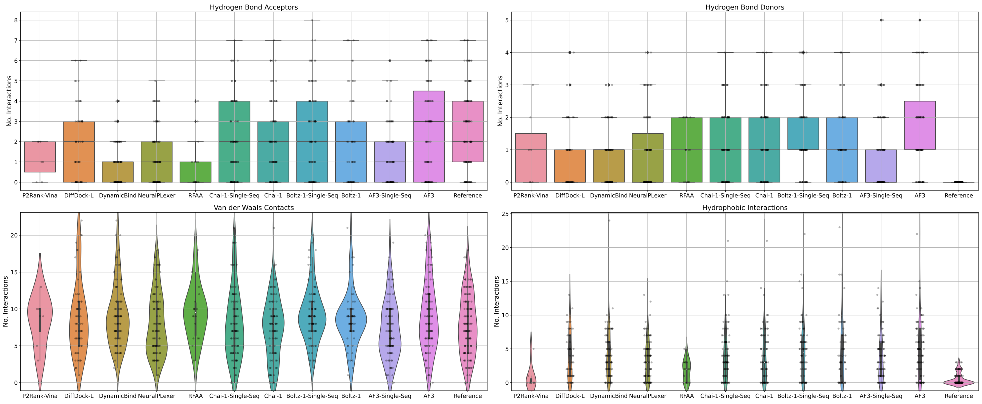

Fig. E8: Comparative analysis of DockGen protein-ligand interactions.

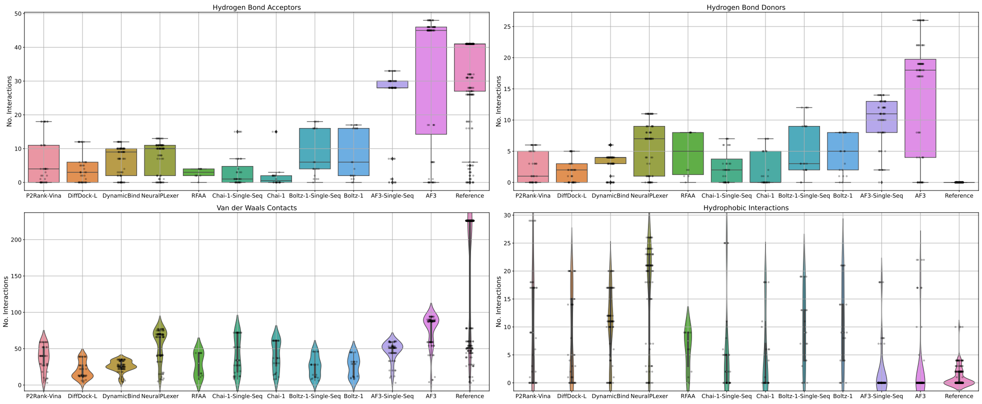

Fig. E9: Comparative analysis of CASP15 protein-ligand interactions.

Appendix F Additional method descriptions

To better contextualize the benchmark's results comparing DL methods to conventional docking algorithms, in this section, we provide further details regarding how each baseline method in the benchmark leverages different sources of biomolecular information to predict PLIs for a given protein target.

F.1 Input and output formats

1. Formats for conventional methods are as follows:
   a) Molecular docking (protein-fixed) software tools such as **AutoDock Vina**, which require specification of protein binding sites, are provided with not only a predicted protein structure from AF3 but also the centroid coordinates of each predicted PLI binding site residue as estimated by the well-known P2Rank binding site prediction algorithm [13]. Such binding site residues are classified using a 10 Å protein-ligand heavy atom interaction threshold and a 25 Å inter-ligand heavy atom interaction threshold to define a "group" of ligands belonging to the same binding site and therefore residing in the same 25 Å^3-sized binding site input voxel for AutoDock Vina. For interested readers, for all four benchmark datasets, we also provide the benchmarking code necessary to run AutoDock Vina using any other baseline method's predicted binding site residues (e.g., those of DiffDock-L) according to the same binding site classification scheme described above.
2. Formats for DL docking methods are as follows:
   a) **DiffDock-L** is provided with a protein structure predicted by AF3 and (fragment) ligand SMILES strings. The model is then tasked with producing (multiple rank-ordered) ligand conformations (for each fragment) for the given protein structure (which remains fixed during docking). Note that DiffDock-L does not natively support multi-ligand SMILES string inputs, so in this work, we propose a modified inference procedure for DiffDock-L which *autoregressively* presents each (fragment) ligand SMILES string to the model while providing the same predicted protein structure to the model in each inference iteration (reporting for each complex the average confidence score over all iterations). Notably, as an inference-time modification, this sampling formulation permits multi-ligand sampling yet cannot model multi-ligand interactions directly and therefore often produces inter-ligand steric clashes.
   b) As a single-ligand DL (flexible) docking method, **DynamicBind** adopts the same input and output formats as DiffDock-L with the following exceptions: (1) the predicted input protein structure is now flexible in response to (fragment) ligand docking; (2) the autoregressive inference procedure we adapted from that of DiffDock-L now provides DynamicBind with its own most recently predicted protein structure in each (fragment) ligand inference iteration, thereby providing the model with partial multi-ligand interaction context; and (3) iteration-averaged confidence scores *and* predicted affinities are reported for each complex. Nonetheless, for both DiffDock-L and DynamicBind, such modified inference procedures highlight the importance in future

work of retraining such generative docking methods directly on multi-ligand complexes to address such inference-time compromises.

3. Formats for DL co-folding methods are as follows:
   a) One of the first DL co-folding methods, **RoseTTAFold-All-Atom** is provided with a (multi-chain) protein sequence as well as (fragment) ligand SMILES strings. The method is subsequently tasked with producing not only a (single) bound ligand conformation but also the bound protein conformation, using diverse MSA databases to provide evolutionary information to the model.
   b) **NeuralPLexer** is a protein-ligand co-folding diffusion model trained using expansive PDB molecule and protein data sources. It receives as its inputs a (multi-chain) protein sequence as well as (fragment) ligand SMILES strings. The method is then tasked with producing multiple rank-ordered (flexible) protein-ligand structure conformations for each input complex, where we use the method's average ligand heavy atom plDDT scores for sampling ranking.
   c) **AlphaFold 3** is a commercially-restricted biomolecular co-folding model trained on exhaustive PDB crystal structures and AlphaFold 2-predicted distillation structures. Following its default settings for inference, the model receives as its inputs a (multi-chain) protein sequence and (fragment) ligand SMILES strings, with default MSA and template inputs provided to the model. The method is then tasked with producing multiple rank-ordered (flexible) protein-ligand structure conformations for each input complex, using the method's intrinsic ranking score [14] for rank-ordering.
   d) **Chai-1** is an open-source co-folding model (akin to AF3) trained on exhaustive PDB crystal structures and AlphaFold 2-predicted distillation structures along with AF3-based training protocols. Following its default settings for inference, the model receives as its inputs a (multi-chain) protein sequence and (fragment) ligand SMILES strings, with paired MSAs yet no template structures provided (as is its default setting). The method is then tasked with producing multiple rank-ordered protein-ligand bound structure conformations for each input complex, using the method's intrinsic AF3-like ranking score for rank-ordering. Note that, as Chai-1's source code does not provide resources to generate multiple sequence alignments for input featurization, Chai-1 uses standardized (taxonomy-paired) multiple sequence alignments akin to those used by AF3 in all benchmarking experiments.
   e) Boltz-1 is an open-source co-folding model (akin to AF3 and Chai-1) trained on exhaustive PDB crystal structures and AlphaFold 2-predicted distillation structures along with AF3-based training protocols. By default, the model receives as its inputs a (multi-chain) protein sequence and (fragment) ligand SMILES strings, with paired MSAs yet no template structures provided (as is its default setting). The method then produces multiple rank-ordered protein-ligand bound structure conformations for each input complex, using the method's intrinsic AF3-like ranking quantity for rank-ordering. Note that, for the sake of benchmarking consistency (n.b., versus Chai-1), Boltz-1 uses standardized (taxonomy-paired) multiple sequence alignments akin to

those used by AF3 in all benchmarking experiments. Note that, by default, we evaluate Boltz-1 with its inference-time potential functions enabled (i.e., Boltz-1x), making its predictions slightly slower but considerably more physically plausible overall (according to the PoseBusters software suite [7]), and we do not evaluate Boltz-2 (trained on PDB data deposited up to June 1, 2023) to maintain the validity of our benchmark's time-splits.

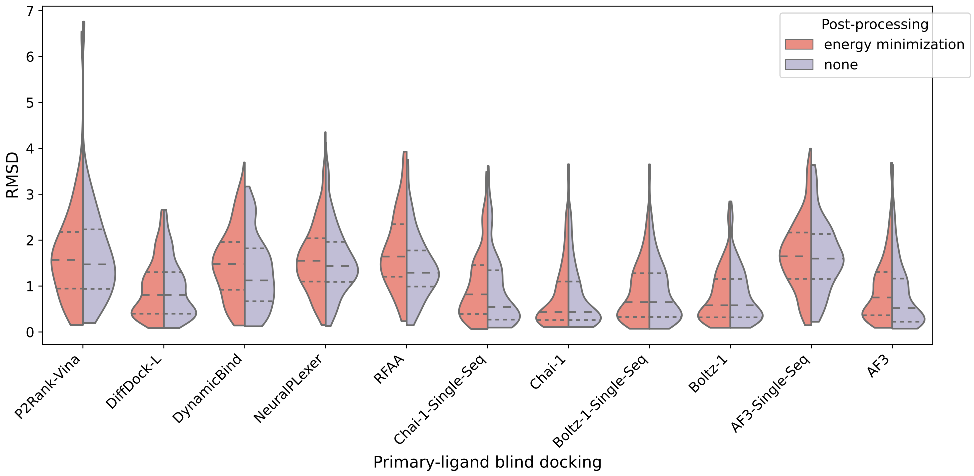

Fig. G10: Astex Diverse dataset results for primary ligand docking RMSD.

Appendix G Additional results

In this section, we provide additional results for each baseline method using the Astex Diverse, PoseBusters Benchmark, and DockGen datasets as well as the CASP15 ligand prediction targets. Note that for all violin plots listed in this section, we curate them using combined results across each method's three independent runs (where applicable), in contrast to this section's bar charts where we instead report mean and standard deviation values across each method's three independent runs.

G.1 Expanded primary ligand results

G.1.1 Primary ligand RMSD results

In Figures G10, G11, and G12, we report the (binding site-superimposed) ligand RMSD values of each baseline method across the primary ligand Astex Diverse, PoseBusters Benchmark, and DockGen datasets, with molecular dynamics (MD)-based structural relaxation applied post-hoc. Overall, these figures demonstrate that Chai-1, Boltz-1, and AF3 achieve the tightest RMSD distributions, except for single-sequence AF3 which occasionally produces catastrophic prediction errors by targeting incorrect PLI binding pockets. Further, these results show that MD-based relaxation generally does not modify the RMSD distribution of most baseline methods, except for DynamicBind and RFAA for which neither seem to benefit from such post-hoc optimizations.

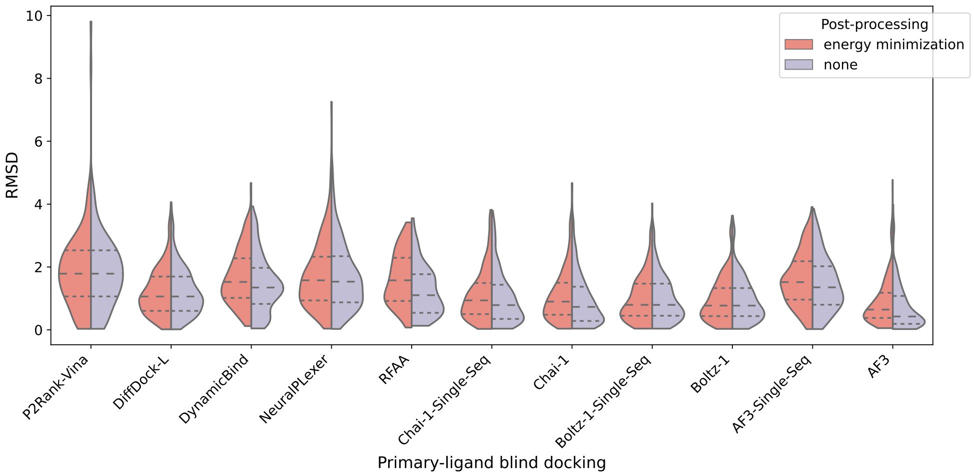

Fig. G11: PoseBusters Benchmark dataset results for primary ligand docking RMSD.

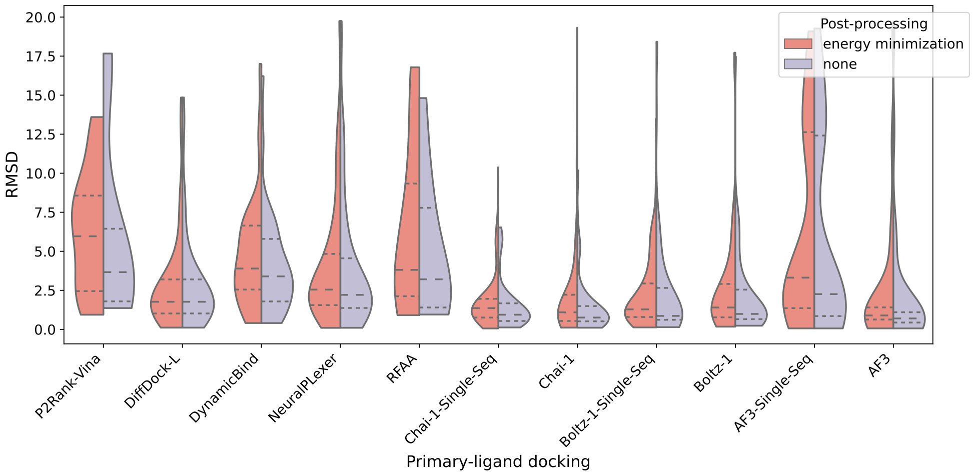

Fig. G12: DockGen dataset results for primary ligand docking RMSD.

G.2 Expanded CASP15 results

G.2.1 Overview of expanded results

In this section, we begin by reporting additional CASP15 benchmarking results in terms of each baseline method's multi-ligand RMSD and lDDT-PLI distributions as violin plots. Subsequently, we report successful ligand docking success rates as well as RMSD and lDDT-PLI results specifically for the single-ligand (i.e., primary ligand) CASP15 targets. Lastly, we report all the above single and multi-ligand results specifically using only the CASP15 targets for which the crystal structures are publicly available, to facilitate reproducible future benchmarking.

G.2.2 Multi-ligand RMSD and lDDT-PLI

To start, Figures G13, G14, and G15 report each method's multi-ligand RMSD and lDDT-PLI distributions as well as PB-Valid rates with and without relaxation. We see that AF3 produces the most tightly bound and accurate RMSD and lDDT-PLI distributions overall yet is challenged in its PB-Valid rate by the conventional method AutoDock Vina, highlighting that AF3 predicted several structurally accurate yet chemically implausible multi-ligand conformations for this dataset.

G.2.3 All single-ligand results

Next, Figures G16, G17, G18, and G19 display each method's single-ligand CASP15 docking success rates, PB-Valid rates, docking RMSD, and docking lDDT-PLI distributions, respectively. In summary, we can make a few respective observations from these plots. (1) Interestingly, Boltz-1 (with and without MSAs) achieves the highest structural and interaction modeling accuracy compared to AF3 and similar co-folding methods. (2) Even though most are positionally incorrect, structurally and chemically speaking, the majority of Boltz-1, AutoDock Vina, and DiffDock-L's predictions are valid according to the PoseBusters software suite, whereas few of AF3's predictions are. (3) Boltz-1 and AF3 yield notably lower RMSD distributions than all other baseline methods (including the similar DL co-folding method Chai-1). (4) Only AutoDock Vina, Boltz-1, and AF3 produce a reasonable range of lDDT-PLI scores for these single-ligand targets.

G.2.4 Single and multi-ligand results for *public* targets

Lastly, for completeness and reproducibility, Figures G20, G21, G22, and G23 present corresponding multi-ligand results for the public CASP15 targets, whereas Figures G24, G25, G26, and G27 report corresponding single-ligand results for the public CASP15 targets. Overall, we observe marginal differences between the full and public CASP15 target results for multi-ligand complexes, since once again AF3 achieves top results overall in the context of multi-ligands. However, for most methods, we notice more striking performance drops between the full and public *single*-ligand CASP15 target results, suggesting that some of the private single-ligand complexes are easier prediction targets than most of the publicly available single-ligand complexes. In short, we find that Boltz-1 consistently performs best in this single-ligand setting.

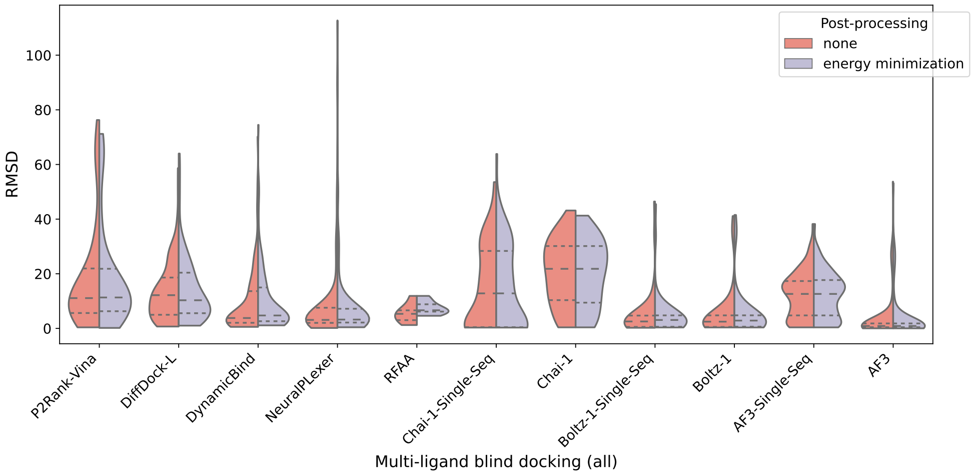

Fig. G13: CASP15 dataset results for multi-ligand docking RMSD with relaxation.

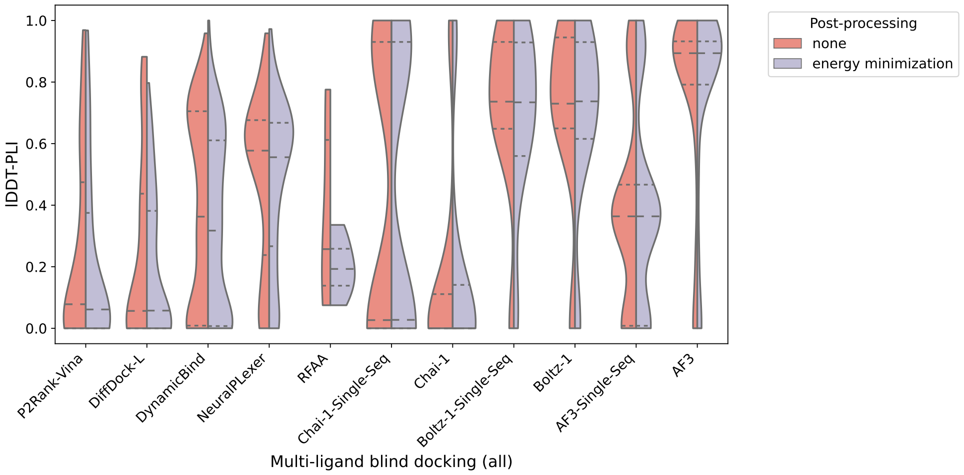

Fig. G14: CASP15 dataset results for multi-ligand docking lDDT-PLI with relaxation.

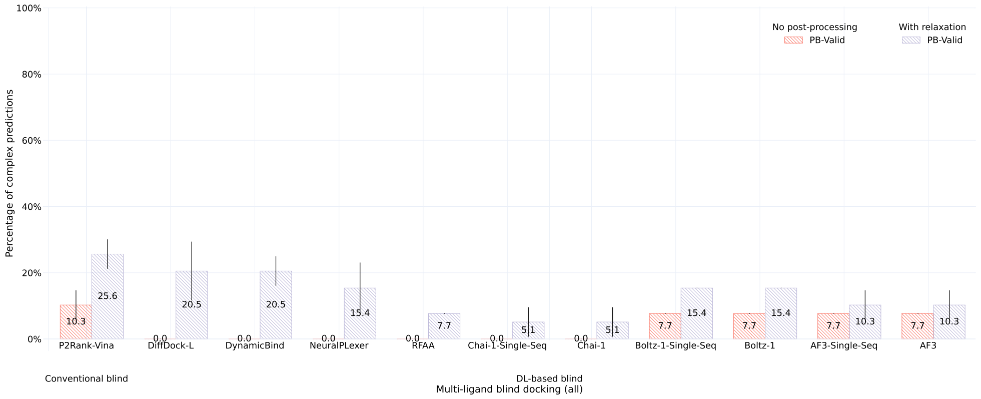

Fig. G15: CASP15 dataset results for multi-ligand docking PB-Valid rates with relaxation. Data are presented as mean values +/- standard deviations over three independent predictions for each protein-ligand complex.

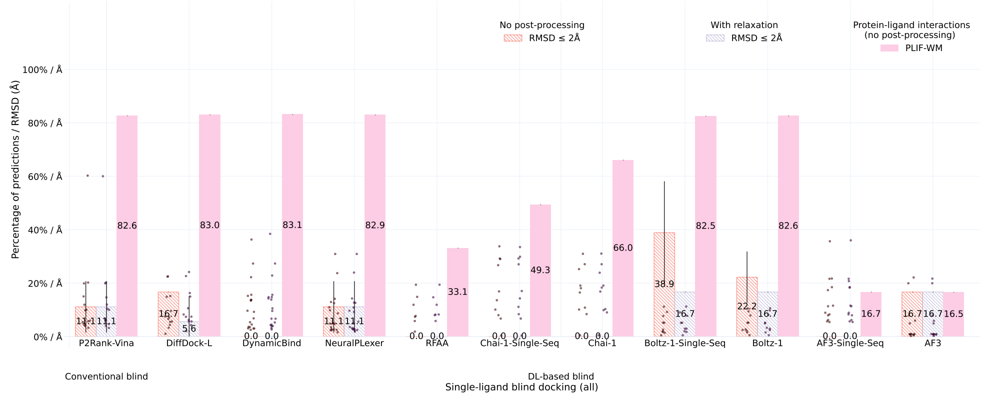

Fig. G16: CASP15 dataset results for successful single-ligand docking with relaxation. Data are presented as mean values +/- standard deviations over three independent predictions for each protein-ligand complex.

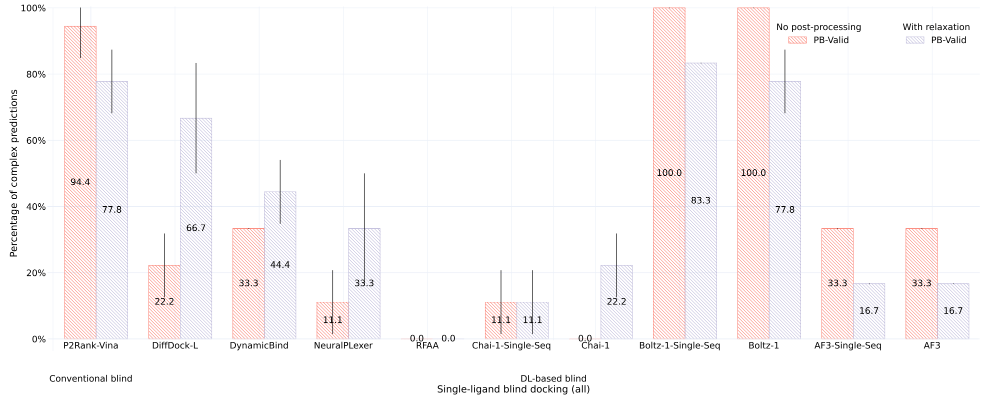

Fig. G17: CASP15 dataset results for single-ligand PB-Valid rates with relaxation. Data are presented as mean values +/- standard deviations over three independent predictions for each protein-ligand complex.

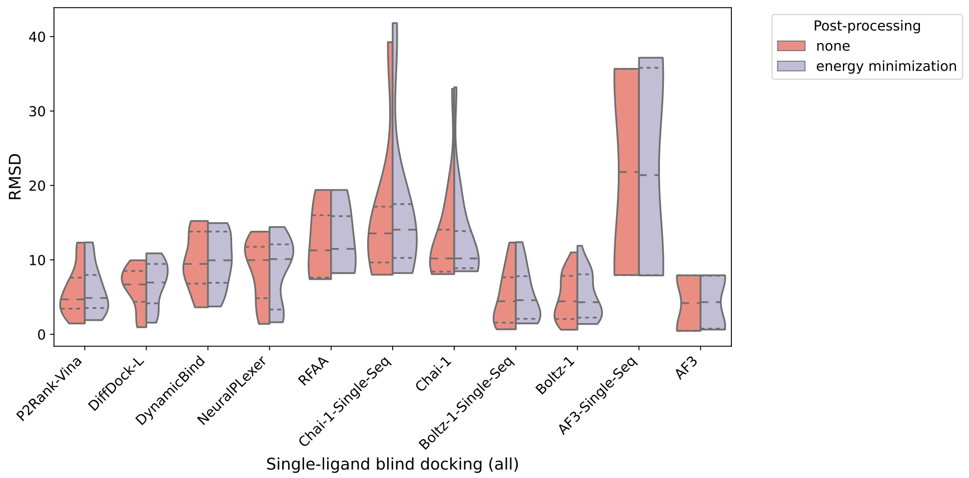

Fig. G18: CASP15 dataset results for single-ligand docking RMSD with relaxation.

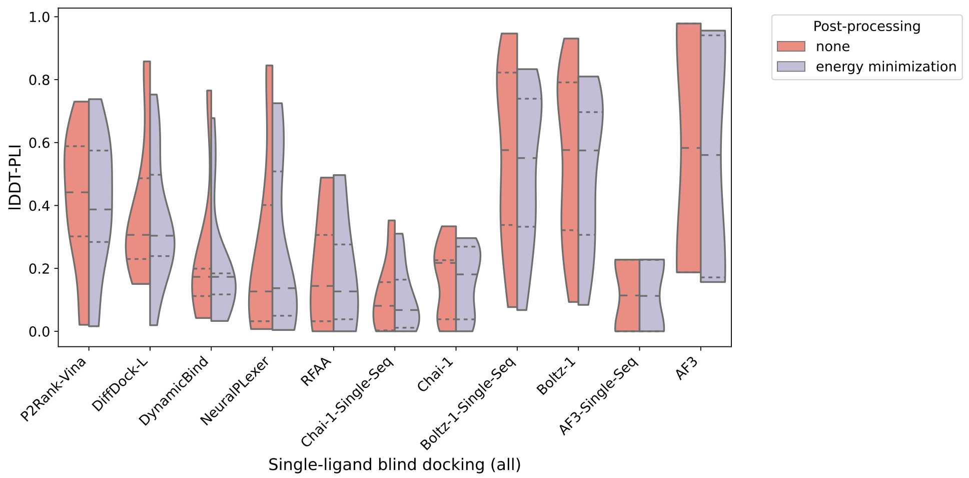

Fig. G19: CASP15 dataset results for single-ligand docking lDDT-PLI with relaxation.

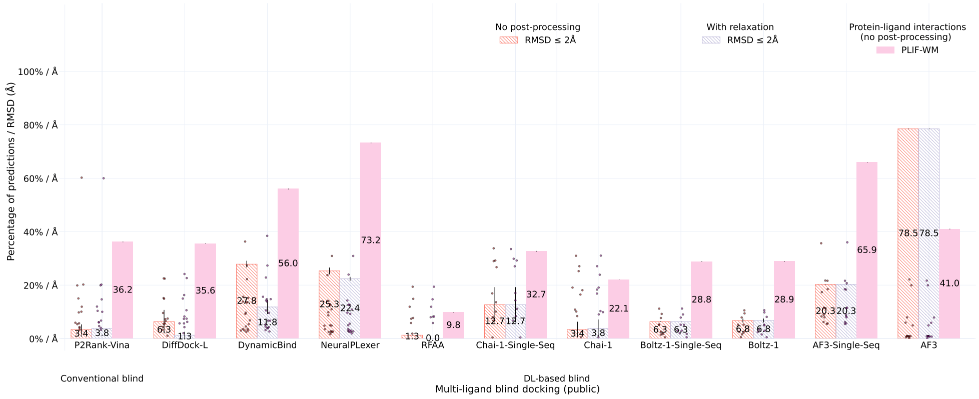

Fig. G20: CASP15 public dataset results for successful multi-ligand docking with relaxation. Data are presented as mean values +/- standard deviations over three independent predictions for each protein-ligand complex.

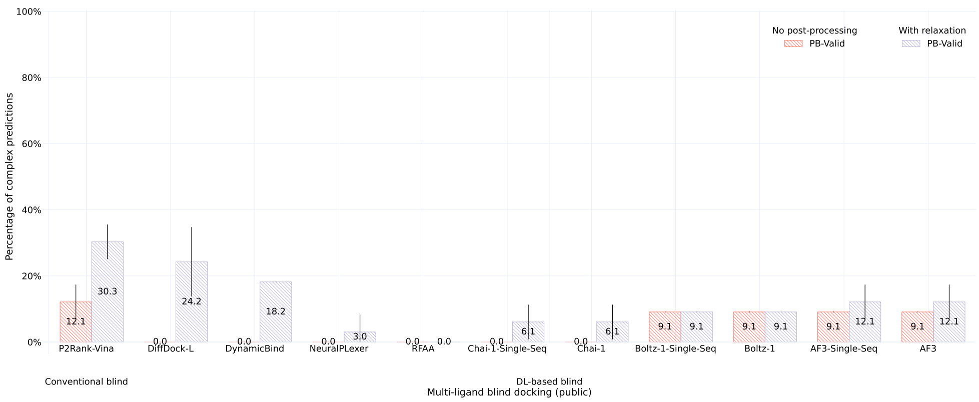

Fig. G21: CASP15 public dataset results for multi-ligand PB-Valid rates with relaxation. Data are presented as mean values +/- standard deviations over three independent predictions for each protein-ligand complex.

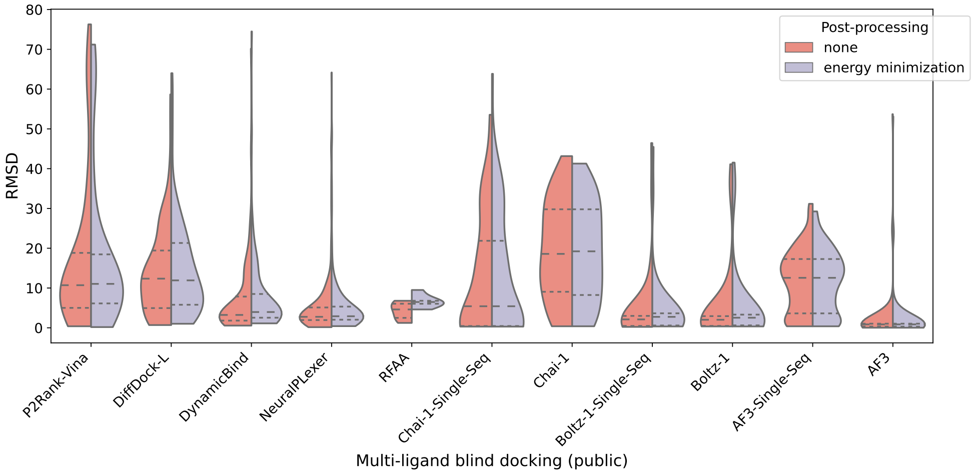

Fig. G22: CASP15 public dataset results for multi-ligand docking RMSD with relaxation.

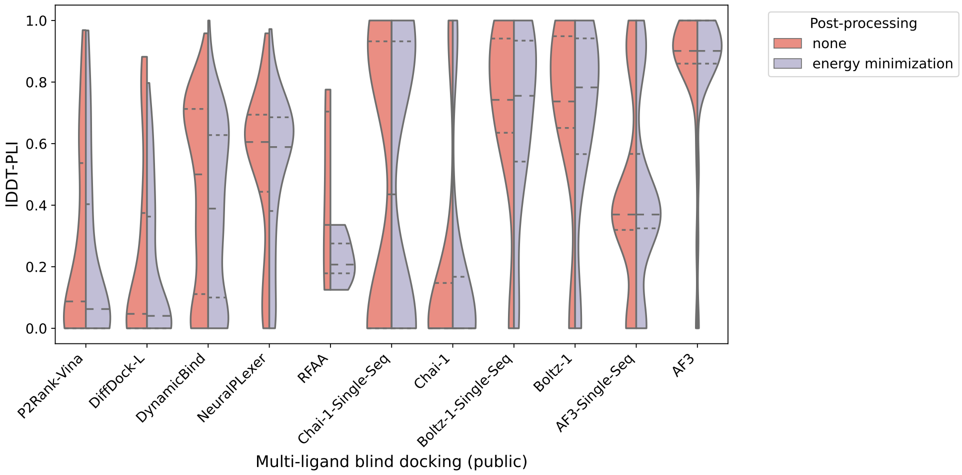

Fig. G23: CASP15 public dataset results for multi-ligand docking lDDT-PLI with relaxation.

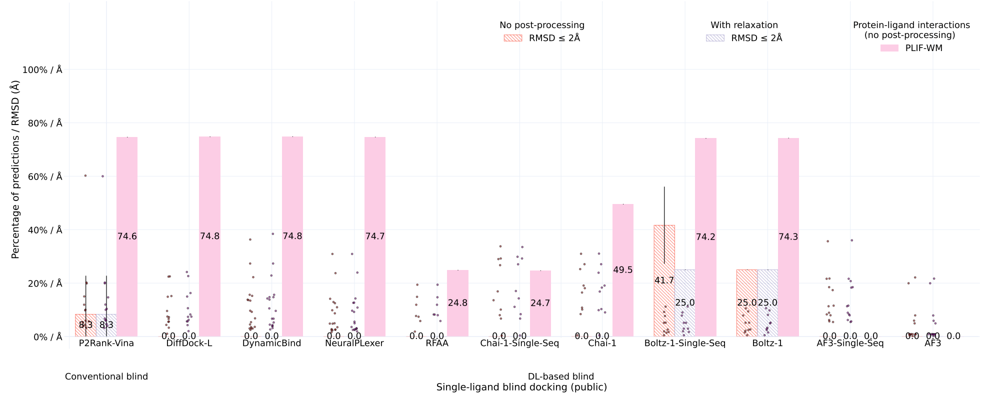

Fig. G24: CASP15 public dataset results for successful single-ligand docking with relaxation. Data are presented as mean values +/- standard deviations over three independent predictions for each protein-ligand complex.

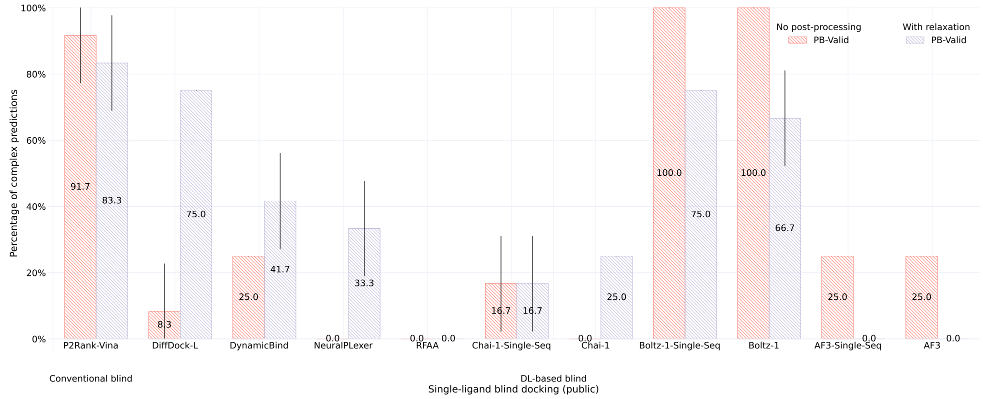

Fig. G25: CASP15 public dataset results for single-ligand PB-Valid rates with relaxation. Data are presented as mean values +/- standard deviations over three independent predictions for each protein-ligand complex.

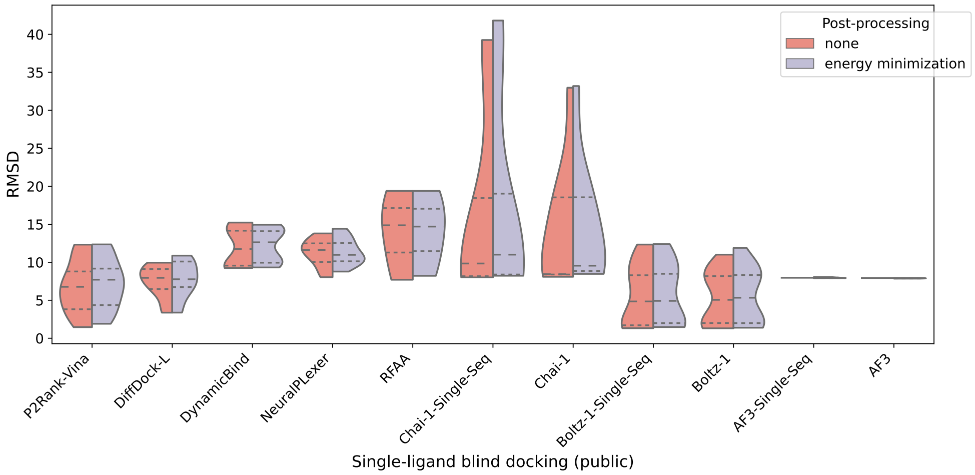

Fig. G26: CASP15 public dataset results for single-ligand docking RMSD with relaxation.

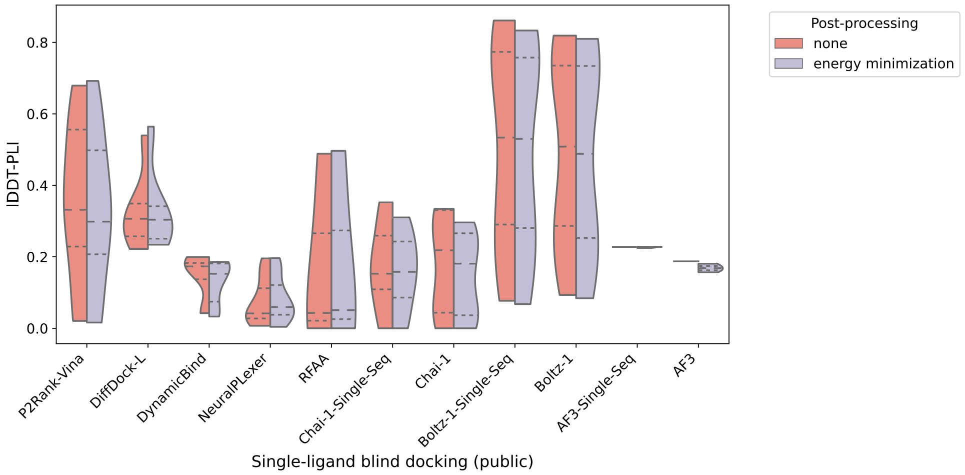

Fig. G27: CASP15 public dataset results for single-ligand docking lDDT-PLI with relaxation.

References

[1] Morehead, A., Giri, N., Liu, J., Neupane, P., Cheng, J.: Assessing the potential of deep learning for protein-ligand docking. Zenodo (2025). https://doi.org/10.5281/zenodo.17536252 . https://doi.org/10.5281/zenodo.17536252

[2] Buttenschoen, M., Morris, G.M., Deane, C.M.: Posebusters: Ai-based docking methods fail to generate physically valid poses or generalise to novel sequences. Chemical Science (2024)

[3] Robin, X., Studer, G., Durairaj, J., Eberhardt, J., Schwede, T., Walters, W.P.: Assessment of protein–ligand complexes in casp15. Proteins: Structure, Function, and Bioinformatics **91**(12), 1811–1821 (2023)

[4] Akhtar, M., Benjelloun, O., Conforti, C., Giner-Miguelez, J., Jain, N., Kuchnik, M., Lhoest, Q., Marcenac, P., Maskey, M., Mattson, P., Oala, L., Ruyssen, P., Shinde, R., Simperl, E., Thomas, G., Tykhonov, S., Vanschoren, J., Vogler, S., Wu, C.-J.: Croissant: A Metadata Format for ML-Ready Datasets (2024)

[5] Hartshorn, M.J., Verdonk, M.L., Chessari, G., Brewerton, S.C., Mooij, W.T., Mortenson, P.N., Murray, C.W.: Diverse, high-quality test set for the validation of protein- ligand docking performance. Journal of medicinal chemistry **50**(4), 726–741 (2007)

[6] Zhang, Y., Skolnick, J.: Scoring function for automated assessment of protein structure template quality. Proteins: Structure, Function, and Bioinformatics **57**(4), 702–710 (2004)

[7] Buttenschoen, M., Morris, G.M., Deane, C.M.: PoseBusters: AI-based docking methods fail to generate physically valid poses or generalise to novel sequences. Zenodo (2023). https://doi.org/10.48550/arXiv.2308.05777 . https://doi.org/10.48550/arXiv.2308.05777

[8] Corso, G., Deng, A., Fry, B., Polizzi, N., Barzilay, R., Jaakkola, T.: Deep confident steps to new pockets: Strategies for docking generalization. arXiv preprint arXiv:2402.18396 (2024)

[9] Corso, G., Deng, A., Fry, B., Polizzi, N., Barzilay, R., Jaakkola, T.: The Discovery of Binding Modes Requires Rethinking Docking Generalization. International Conference on Learning Representations (ICLR) (2024). https://doi.org/10.5281/zenodo.10656052 . https://doi.org/10.5281/zenodo.10656052

[10] Stärk, H., Jing, B., Barzilay, R., Jaakkola, T.: Harmonic self-conditioned flow matching for multi-ligand docking and binding site design. arXiv preprint arXiv:2310.05764 (2023)

[11] Bank, P.D.: Protein data bank. Nature New Biol **233**(223), 10–1038 (1971)

[12] Harris, C., Didi, K., Jamasb, A.R., Joshi, C.K., Mathis, S.V., Lio, P., Blundell, T.: Benchmarking generated poses: How rational is structure-based drug design with generative models? arXiv preprint arXiv:2308.07413 (2023)

[13] Kriválk, R., Hoksza, D.: P2rank: machine learning based tool for rapid and accurate prediction of ligand binding sites from protein structure. Journal of cheminformatics **10**, 1–12 (2018)

[14] Abramson, J., Adler, J., Dunger, J., Evans, R., Green, T., Pritzel, A., Ronneberger, O., Willmore, L., Ballard, A.J., Bambrick, J., et al.: Accurate structure prediction of biomolecular interactions with alphafold 3. Nature, 1–3 (2024)
