# Supplementary material for: Assessing the potential of deep learning for protein–ligand docking
Source: Nat Mach Intell. 2025 Dec 31;8(1):32–41. doi: 10.1038/s42256-025-01160-1 (PMC12851923; doi:10.1038/s42256-025-01160-1)
Supplement: Supplementary file 2 — Reporting Summary [file 42256_2025_1160_MOESM2_ESM.pdf]

nature portfolio

Corresponding author(s): Alex Morehead
Last updated by author(s): Nov 4, 2025

Reporting Summary

Nature Portfolio wishes to improve the reproducibility of the work that we publish. This form provides structure for consistency and transparency in reporting. For further information on Nature Portfolio policies, see our Editorial Policies and the Editorial Policy Checklist.

Statistics

For all statistical analyses, confirm that the following items are present in the figure legend, table legend, main text, or Methods section.

n/a	Confirmed	
☐	☒	The exact sample size (*n*) for each experimental group/condition, given as a discrete number and unit of measurement
☐	☒	A statement on whether measurements were taken from distinct samples or whether the same sample was measured repeatedly
☐	☒	The statistical test(s) used AND whether they are one- or two-sided<br>*Only common tests should be described solely by name; describe more complex techniques in the Methods section.*
☒	☐	A description of all covariates tested
☒	☐	A description of any assumptions or corrections, such as tests of normality and adjustment for multiple comparisons
☐	☒	A full description of the statistical parameters including central tendency (e.g. means) or other basic estimates (e.g. regression coefficient) AND variation (e.g. standard deviation) or associated estimates of uncertainty (e.g. confidence intervals)
☐	☒	For null hypothesis testing, the test statistic (e.g. *F*, *t*, *r*) with confidence intervals, effect sizes, degrees of freedom and *P* value noted<br>*Give P values as exact values whenever suitable.*
☒	☐	For Bayesian analysis, information on the choice of priors and Markov chain Monte Carlo settings
☒	☐	For hierarchical and complex designs, identification of the appropriate level for tests and full reporting of outcomes
☐	☒	Estimates of effect sizes (e.g. Cohen's *d*, Pearson's *r*), indicating how they were calculated

Our web collection on statistics for biologists contains articles on many of the points above.

Software and code

Policy information about availability of computer code

Data collection	All data collection code can be found at https://github.com/BioinfoMachineLearning/PoseBench. Specifically, the code makes use of the Python packages hydra-core 1.3.2, biopandas 0.5.1.dev0, biopython 1.79, meeko 0.6.0a3, numpy 1.26.4, pandas 1.5.0, posebusters 0.4.5, posecheck 1.1, prolif 2.0.3, pypdb 2.3, rdkit 2025.3.5, scikit-learn 1.1.2, seaborn 0.12.2, and spyrmsd 0.5.2.
Data analysis	All data analysis code can be found at https://github.com/BioinfoMachineLearning/PoseBench. Specifically, the code makes use of the Python packages hydra-core 1.3.2, biopandas 0.5.1.dev0, biopython 1.79, meeko 0.6.0a3, numpy 1.26.4, pandas 1.5.0, posebusters 0.4.5, posecheck 1.1, prolif 2.0.3, pypdb 2.3, rdkit 2025.3.5, scikit-learn 1.1.2, seaborn 0.12.2, and spyrmsd 0.5.2.

For manuscripts utilizing custom algorithms or software that are central to the research but not yet described in published literature, software must be made available to editors and reviewers. We strongly encourage code deposition in a community repository (e.g. GitHub). See the Nature Portfolio guidelines for submitting code & software for further information.

Data

Policy information about availability of data

All manuscripts must include a data availability statement. This statement should provide the following information, where applicable:

- Accession codes, unique identifiers, or web links for publicly available datasets
- A description of any restrictions on data availability
- For clinical datasets or third party data, please ensure that the statement adheres to our policy

The PoseBench datasets and benchmark results are available at https://zenodo.org/records/17536252 under a Creative Commons Attribution 4.0 International Public License, with further licensing discussed in Appendix A and detailed dataset documentation provided in Appendix D.

Research involving human participants, their data, or biological material

Policy information about studies with human participants or human data. See also policy information about sex, gender (identity/presentation), and sexual orientation and race, ethnicity and racism.

Reporting on sex and gender	N/A
Reporting on race, ethnicity, or other socially relevant groupings	N/A
Population characteristics	N/A
Recruitment	N/A
Ethics oversight	N/A

Note that full information on the approval of the study protocol must also be provided in the manuscript.

Field-specific reporting

Please select the one below that is the best fit for your research. If you are not sure, read the appropriate sections before making your selection.

☒ Life sciences ☐ Behavioural & social sciences ☐ Ecological, evolutionary & environmental sciences

For a reference copy of the document with all sections, see nature.com/documents/nr-reporting-summary-flat.pdf

Life sciences study design

All studies must disclose on these points even when the disclosure is negative.

Sample size	A total of 356 protein–ligand complexes were studied across four well-known benchmark datasets containing 85, 122, 130, and 13+6 complexes, respectively. These datasets were selected to align with the scope of the PoseBench benchmark, which combines existing publicly available protein–ligand datasets with newly introduced multi-ligand sets. Each dataset was previously published or documented for its utility in revealing the behavior of protein–ligand docking methods across diverse biological contexts, enabling clear demarcation and extension. Despite the scarcity of high-quality, publicly available complexes, this collection represents a non-overlapping mix of single- and multi-ligand cases, spanning high to low interaction similarity relative to common machine learning training data. The diversity and number of complexes are sufficient to conduct statistical significance testing and to rigorously evaluate machine learning-based docking methods under multiple scenarios, including apo-to-holo docking, multi-ligand binding, structure recall, and low-similarity generalization.
Data exclusions	178 protein-ligand complexes from the PoseBusters Benchmark dataset were excluded since they overlap with the training dataset of AlphaFold 3 and Boltz-1.
Replication	We have rerun each experiment included in this study three times and report the mean and standard deviation of each experiment. All attempts at replication were successful.
Randomization	This study used publicly available protein-ligand datasets curated for benchmark evaluation. Samples (protein–ligand complexes) were allocated into experimental conditions based on predefined task categories (e.g., apo-to-holo docking, multi-ligand docking) rather than by random assignment. As the datasets are fixed and not subject to biological variation, covariate control was not applicable. All algorithms were evaluated on identical datasets to ensure fair comparison.
Blinding	This study involved only computational experiments on publicly available protein-ligand datasets, with no human or animal subjects. Data collection consisted of running predefined algorithms on these datasets, and analysis involved automated evaluation against known structures. As group allocation is not applicable in this context, blinding was not relevant to the study design.

Reporting for specific materials, systems and methods

We require information from authors about some types of materials, experimental systems and methods used in many studies. Here, indicate whether each material, system or method listed is relevant to your study. If you are not sure if a list item applies to your research, read the appropriate section before selecting a response.

Materials & experimental systems

n/a	Involved in the study
☒	☐ Antibodies
☒	☐ Eukaryotic cell lines
☒	☐ Palaeontology and archaeology
☒	☐ Animals and other organisms
☒	☐ Clinical data
☒	☐ Dual use research of concern
☒	☐ Plants

Methods

n/a	Involved in the study
☒	☐ ChIP-seq
☒	☐ Flow cytometry
☒	☐ MRI-based neuroimaging

Plants

Seed stocks	N/A
Novel plant genotypes	N/A
Authentication	N/A
